# Imaging and functional correlates of fibrosis in neovascular age-related macular degeneration: a systematic review

**DOI:** 10.3389/fopht.2026.1786309

**Published:** 2026-02-24

**Authors:** Kimberly L. Spooner, Samantha Fraser-Bell, Dun Jack Fu, Livia Faes, Francesco Romano, Mariano Cozzi, Andrew A. Chang, Sobha Sivaprasad

**Affiliations:** 1 Save Sight Institute, The University of Sydney, Sydney, NSW, Australia; 2 The Graduate School of Health, The University of Technology, Sydney, NSW, Australia; 3 Sydney Eye Hospital, Sydney, NSW, Australia; 4 Royal North Shore Hospital, St. Leonards, NSW, Australia; 5 Institute of Ophthalmology, University College London, London, United Kingdom; 6 National Institute for Health Research Biomedical Research Centre, Moorfields Eye Hospital National Health Service Foundation Trust, London, United Kingdom; 7 Eye Clinic, Department of Biomedical and Clinical Sciences, Luigi Sacco Hospital, University of Milan, Milan, Italy; 8 Department of Ophthalmology, University Hospital Zurich, University of Zurich, Zurich, Switzerland; 9 Sydney Retina Clinic, Sydney, NSW, Australia

**Keywords:** age-related macular degeneration, anti-VEGF therapy, imaging biomarkers, optical coherence tomography, subretinal fibrosis, visual function

## Abstract

**Background:**

Despite intravitreal anti–vascular endothelial growth factor (VEGF) therapy being the standard of care for neovascular age-related macular degeneration (nAMD), long-term visual decline remains common, with subretinal fibrosis representing a major cause of irreversible vision loss. Objective: To systematically evaluate how imaging-defined fibrosis in nAMD is defined and quantified, its incidence under anti-VEGF therapy, associated baseline associations, and its impact on visual outcomes.

**Methods:**

We systematically searched MEDLINE, Embase, CENTRAL, and Scopus through September 2025 for studies reporting imaging-defined fibrosis in anti-VEGF–treated nAMD. Eligible studies included randomized controlled trial secondary analyses, prospective and retrospective cohorts, and registries. Two reviewers independently extracted data on fibrosis definitions, imaging modalities, associations, and functional outcomes. Random-effects meta-analyses pooled the best-corrected visual acuity (BCVA) difference (ETDRS letters) and the odds ratio for incident fibrosis. Risk of bias was assessed using the Quality in Prognosis Studies (QUIPS) tool, and the certainty of evidence was evaluated using the GRADE approach.

**Results:**

Fifty-eight studies were included (12 randomized trial secondary analyses, 18 prospective studies, and 28 retrospective studies). Across studies, subretinal fibrosis developed in approximately 10–15% of eyes within 2 years and 40–50% by 5 years of anti-VEGF therapy, with a lower incidence under fixed or treat-and-extend regimens compared with pro re nata dosing. Eyes with fibrosis had consistently worse visual outcomes (pooled BCVA difference −29 ETDRS letters; 95% CI −47 to −12). Key associations included type 2 macular neovascularisation (OR 5.7), subretinal hyperreflective material (OR 2.7), intraretinal fluid (OR 3.6), and large haemorrhage (OR 2.3), while subretinal fluid appeared protective (OR 0.6). Definitions and quantification approaches varied widely across imaging modalities.

**Conclusions:**

Fibrosis remains a frequent and vision-limiting sequela of treated nAMD, with substantial heterogeneity in imaging definitions and grading methods limiting cross-study comparability. Standardised OCT-anchored definitions, reproducible quantitative measures, and functional endpoints beyond BCVA are needed to advance anti-fibrotic therapeutic development and improve long-term visual outcomes.

**Systematic Review Registration:**

https://www.crd.york.ac.uk/prospero/, identifier CRD420231132016.

## 1 Introduction

Histopathological studies have long shown that subretinal fibrosis in neovascular age-related macular degeneration (nAMD) represents an aberrant wound-healing response, characterized by infiltration of myofibroblasts, extracellular matrix deposition, and disruption of the retinal pigment epithelium (RPE) and photoreceptors (1, 2). This biological process often culminates in the disorganization of the outer retina and irreversible loss of central vision. Over the past two decades, intravitreal anti-vascular endothelial growth factor (VEGF) therapy has transformed nAMD from a relentlessly blinding condition into one where long-term visual preservation is achievable for many patients (3, 4). This therapeutic revolution has changed the natural history of the disease and significantly reduced rates of severe vision loss (5, 6).

Despite these advances, many eyes ultimately lose vision not from uncontrolled exudation, but from the gradual development of subretinal and sub-RPE fibrosis (7–9). Fibrotic scar formation represents the culmination of a maladaptive wound-healing response, resulting in structural disorganization, loss of photoreceptors, and irreversible decline in visual function (10, 11). Crucially, no current therapy effectively prevents or reverses this process, making fibrosis one of the most significant unmet needs in the long-term management of nAMD.

The reported incidence of fibrosis varies widely depending on baseline characteristics, treatment regimen, imaging modality, and follow-up duration. Data from pivotal trials and registries suggest that 10–25% of eyes develop fibrosis within the first two years of therapy, rising to 40–50% at five years and nearly 50% at ten years (8, 12, 13). These reports are mainly based on fibrosis diagnosed on colour fundus photographs (CFP). For example, fibrosis accounts for a significant portion of the late visual decline observed in the CATT and IVAN Cohorts, even among patients who initially experienced vision improvement under anti-VEGF treatment. These observations indicate that the process of fibrosis may occur earlier than visually perceived on CFP. On CFP, fibrosis corresponds to the development of well-demarcated fibrotic scars, distinguishable from nonfibrotic scars by their elevated, whitish appearance and associated loss of retinal detail, as described in the CATT study (14).

However, earlier changes are evident on optical coherence tomography (OCT), where the transformation of subretinal hyperreflective material (SHRM) into dense, hyperreflective scar tissue is often accompanied by RPE disruption (15, 16). On fundus autofluorescence (FAF), fibrotic lesions appear as areas of hypo- or hyper-autofluorescence and are usually challenging to decipher from other components of the neovascular complex (17, 18). In contrast, fundus fluorescein angiography (FFA) describes late-staining fibrotic plaques (19). More advanced modalities, such as polarisation-sensitive (PS)-OCT, can detect birefringence signals specific to fibrotic tissue (15, 20, 21), and OCT angiography may demonstrate flow voids over scarred areas, the significance of which remains poorly understood (22, 23). Multimodal imaging strategies provide greater diagnostic confidence but remain resource-intensive. The lack of a standardized, universally adopted definition impedes cross-study comparability and limits consistent tracking of fibrotic progression.

Quantification of fibrosis is similarly heterogeneous. Most studies rely on categorical grading (present/absent, fibrotic vs. nonfibrotic), while others use manual area measurements from fundus photographs or *en face* OCT images. (23, 24) Volumetric approaches have been explored using polarisation-sensitive OCT and experimental AI-based segmentation methods (21, 24, 25), although these remain unvalidated in large-scale clinical trials and real-world settings.

Clinically, fibrosis is strongly associated with worse best-corrected visual acuity (BCVA) and is one of the most significant predictors of long-term functional decline in treated nAMD (8). Less is known about its relationship to other functional endpoints, such as contrast sensitivity, microperimetry, or patient-reported outcomes. However, emerging evidence suggests that fibrosis is associated with reduced retinal sensitivity and visual function, thereby impairing quality of life (15, 17).

However, fibrosis in nAMD is not defined uniformly across studies. Reported definitions range from colour fundus photograph–defined scars to OCT-derived hyperreflective material, sub-RPE fibrovascular tissue, and birefringent collagen detected on polarization-sensitive OCT. These constructs are related but not interchangeable, complicating the synthesis and interpretation of the literature. This systematic review aimed to evaluate how imaging-derived fibrosis in nAMD is defined and quantified across imaging modalities, and to synthesize the evidence on associated outcomes within this heterogeneity. Specifically, we sought to characterize (i) definitions and quantification methods, (ii) the incidence and time course of fibrosis under anti-VEGF therapy, and (iii) its association with BCVA and other functional endpoints.

## 2 Methods

### 2.1 Study design

This systematic review was conducted in accordance with the Preferred Reporting Items for Systematic Reviews and Meta-Analyses (PRISMA) 2020 guidelines (26). This review was prospectively registered in the PROSPERO database (registration number CRD42023 1132016).

### 2.2 Eligibility criteria

We included studies of adults with nAMD treated with intravitreal anti-VEGF therapy, in which fibrosis was defined and/or its incidence assessed using recognized imaging modalities (OCT, FAF, FA/CFP, PS-OCT, or multimodal imaging). Studies employing OCT-angiography (OCT-A) were included when fibrosis was correlated with vascular network characteristics (e.g., macular neovascularisation (MNV) morphology, flow voids, or non-perfusion), rather than direct structural definition. Eligible comparators were eyes without fibrosis, or studies comparing different fibrosis subtypes (e.g., subretinal vs sub-RPE). Regarding functional endpoints, the primary outcome was BCVA, measured in the Early Treatment diabetic retinopathy study (ETDRS) letters or logMAR, at ≥12 months. Secondary functional outcomes included contrast sensitivity (CS) (Pelli-Robson chart), low-luminance visual acuity (LLVA) (ETDRS chart under a 2.0 log neutral density filter), reading speed (MNRead chart), microperimetry, and patient-reported outcomes (PROs) (NEI-VFQ-25), where available. Eligible designs comprised prospective or retrospective cohort studies, registry-based analyses, and secondary analyses of randomized controlled trials (RCTs). We excluded non-human studies, case reports or small case series (<10 eyes), reviews, editorials, conference abstracts, and studies lacking imaging-defined fibrosis or functional outcomes.

### 2.3 Search methods for identifying studies

We systematically searched MEDLINE (PubMed), Embase, Cochrane CENTRAL, and Scopus from January 2015 to September 2025. Search terms combined controlled vocabulary and free-text keywords related to age-related macular degeneration, fibrosis, scar, imaging, and functional outcomes. The full search strategies are provided in **Supplementary Appendix 1**.

### 2.4 Study selection and data collection

**Figure 1:**
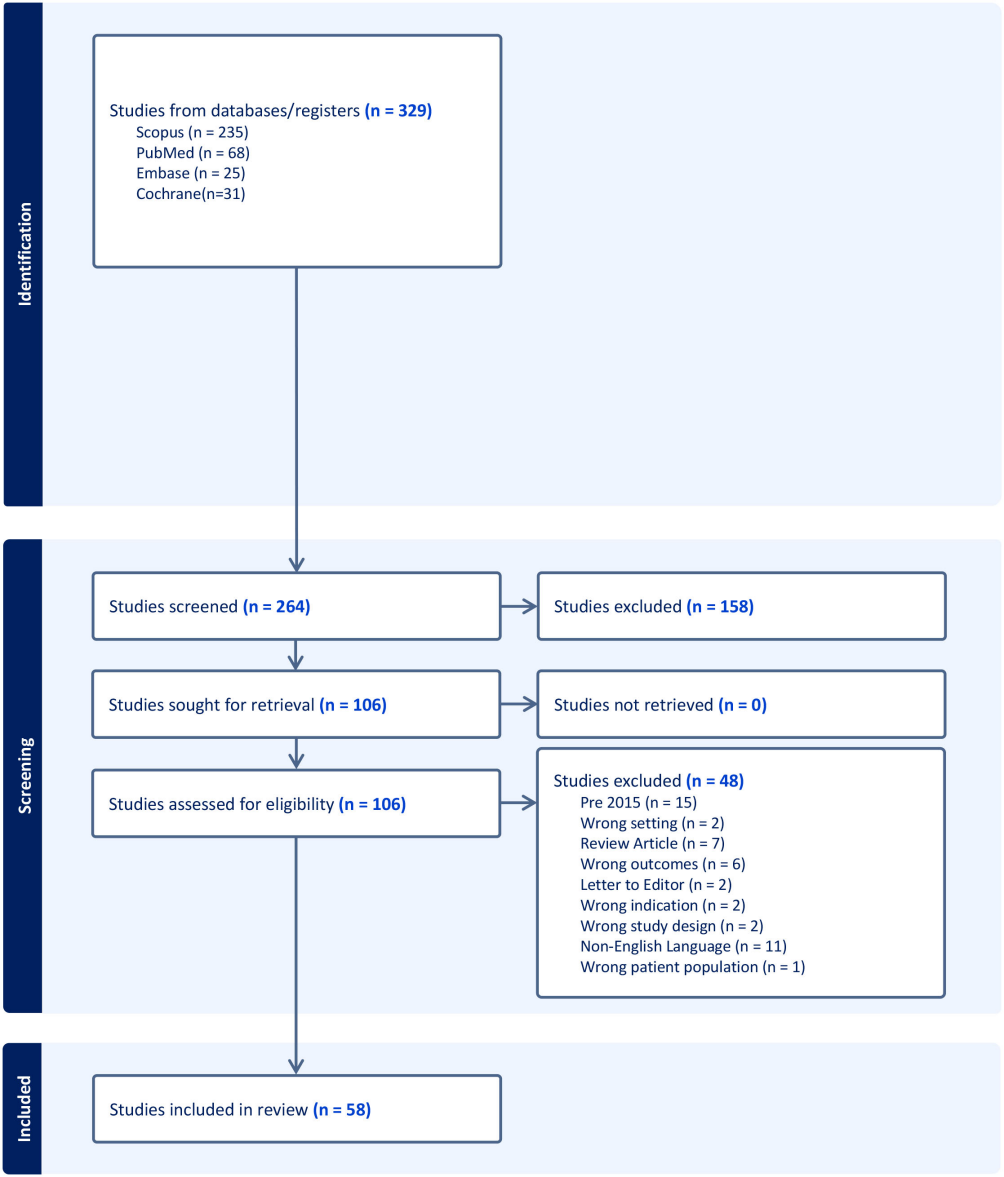
PRISMA flow diagram depicting the study selection process for the systematic review. The diagram illustrates the number of records identified, screened, excluded, and included, with reasons for exclusion at each stage.

Two reviewers independently screened all titles and abstracts, followed by a full-text review of potentially eligible studies. Discrepancies were resolved through discussion or by a third-party arbitrator. Duplicates across databases were removed using Covidence and confirmed by manual review before screening. Study selection is presented in a PRISMA flow diagram (**Figure 1**).

Data were extracted using a standardized form for study design, sample size, follow-up duration, anti-VEGF regimen, patient demographics, imaging modality, fibrosis definition and quantification method, and reported outcomes (BCVA mean ± SD, odds ratios [OR] or hazard ratios [HR], or regression coefficients for poor vision, and secondary outcomes). Two reviewers independently extracted data, and discrepancies were resolved by consensus.

### 2.5 Risk of bias assessment

The risk of bias was assessed using the Quality in Prognosis Studies (QUIPS) tool (27), evaluating six domains: study participation, study attrition, measurement of prognostic factors, measurement of outcomes, confounding, and statistical analysis.

### 2.6 Data synthesis and statistical analysis

Meta-analyses were conducted when studies reported comparable outcome constructs (e.g., fibrosis present versus absent), effect measures (odds ratios or hazard ratios), and variance estimates, even when imaging-based definitions of fibrosis differed. Given the absence of standardized definitions of fibrosis across imaging modalities, meta-analyses were undertaken as exploratory, hypothesis-generating syntheses. Pooling was intended to evaluate consistency in the direction of associations across heterogeneous definitions, rather than to estimate precise or interchangeable effect sizes. We anticipated substantial residual clinical heterogeneity due to differences in imaging definitions, grading thresholds, and follow-up duration, which could not be fully resolved statistically. Continuous outcomes, such as BCVA, were pooled as mean differences with 95% confidence intervals (CI) using inverse-variance weighting under a random-effects model (DerSimonian-Laird method). Dichotomous outcomes were expressed as pooled odds ratios or hazard ratios.

Between-study heterogeneity was quantified using the I², τ², and Cochran’s Q statistics. Publication bias was assessed using Egger’s regression and Begg’s rank correlation tests, and funnel plots were visually inspected.

Pre-specified subgroup analyses included MNV subtypes according to the CONAN recommendations (Types 1–3 and polypoidal choroidal vasculopathy, PCV), treatment regimen (fixed, PRN, treat-and-extend), and study design (randomized controlled trial, RCT vs. real-world). Sensitivity analyses excluded high-risk-of-bias studies.

Narrative synthesis was used when quantitative pooling was inappropriate, grouping studies by imaging modality, fibrosis definition, and follow-up duration.

### 2.7 Certainty of evidence

The overall certainty of evidence was evaluated using the GRADE framework adapted for prognostic studies (28, 29), considering risks of bias, inconsistency, indirectness, imprecision, and publication bias. GRADE profiles were prepared to inform clinical interpretation of key biomarker-outcome associations.

## 3 Results

### 3.1 Study selection and characteristics

A total of 58 studies met the inclusion criteria and were incorporated into the synthesis (PRISMA flow shown in **Figure 1**). Reasons for exclusion are detailed in **Supplementary Table S1**. These comprised 13 secondary analyses of randomized controlled trials (9, 15, 16, 19, 24, 30–34), 28 prospective cohort studies (8, 17, 18, 21–23, 25, 35–52), and 17 retrospective cohorts (12, 16, 53–71) Sample sizes varied widely, from as few as 15 eyes to more than 1,200, with the most extensive datasets derived from the landmark CATT and IVAN trials and Fight Retinal Blindness! (FRB) registries (12, 19, 49, 72). The follow-up duration ranged from 12 months to 10 years, with a median of approximately 36 months across the studies. The included evidence spanned North America, Europe, the Asia-Pacific region, and several multicenter registries.

Study-level characteristics, including design, sample size, follow-up, imaging modality, definitions of fibrosis, and outcomes assessed, are summarised in **Table 1**.

**Table 1:** Baseline characteristics of included studies (n=58).

Study (Author, Year)	Country / Setting	Study design	Sample size (eyes)	Treatment regimen	Imaging modality	Fibrosis definition	Follow-up (months)	Outcome measure(s)
Angermann et al., 2022 (36)	Austria (Innsbruck, Salzburg)	Retrospective cohort	648 eyes	Aflibercept treat-and-extend	OCT, CF	Subfoveal fibrosis (end-stage, compact HRM)	60	BCVA (logMAR), fibrosis incidence (persistent vs nonpersistent)
Barikian et al., 2015 (53)	Lebanon, Qatar, UAE (Single-institution)	Prospective randomized pilot study	90 (30 per group)	Bevacizumab q2w ×3 vs q4w ×3 vs immediate PRN	OCT, FA	Subretinal fibrosis (clinical/OCT based)	12	Fluid-free interval, BCVA, CRT, subretinal fibrosis
Bilgic et al., 2021 (54)	Multicentre (India, France, Germany)	Retrospective observational	458 screened; 63 eyes complete resolution	Ranibizumab / Aflibercept	SD-OCT	Fibrosis = collagenous tissue buildup in retina/subretinal/sub-RPE space (per Spaide consensus)	≥24	BCVA, CRT, exudation resolution, fibrosis absence/presence
Cao et al., 2024 (37)	China (Xi’an)	Cross-sectional	16 fibrosis eyes, 33 non-fibrosis, 28 controls	Prior anti-VEGF	OCT, FA, ICGA, OCTA, aqueous cytokine assays	Subretinal fibrosis (clinical + multimodal criteria)	Cross-sectional	Aqueous cytokines, BCVA (logMAR), SHRM thickness
Casalino et al., 2018 (35)	UK (single tertiary referral centre)	Retrospective observational study	150	Ranibizumab (PRN after loading) or aflibercept (fixed q8w for 1y)	OCT, CF, NIR, FA, ICG	Macular scar classified as fibrotic vs nonfibrotic (CATT criteria)	12	HRM morphology, fibrotic scar, MA, BCVA
Chakravarthy et al., 2015 (IVAN trial) (19)	UK, 23 NHS hospitals	Multicentre RCT (factorial: drug × regimen)	610 (1 eye per pt)	Ranibizumab 0.5 mg vs bevacizumab 1.25 mg; continuous monthly vs discontinuous PRN	OCT, FA, CFP	Fibrotic scar per trial reading centre (CATT/IVAN def.)	24	BCVA (ETDRS), lesion morphology, GA, PROs
Chandak et al., 2025 (PRECISE 7) (38)	UK multicentre	Retrospective registry (loading dose cohort)	1999 eyes	Aflibercept 2 mg × 3 (loading phase)	SD-OCT	Fibrosis recorded as OCT feature predictive of poor VA	3 (loading)	BCVA ≥68 letters, <54 letters, logistic regression
Chandra et al., 2024 (PRECISE Report 2) (68)	UK (10 centres)	Predictive modelling (cross-sectional analysis)	2039	Aflibercept loading (3 monthly)	OCT (Spectralis)	Foveal fibrosis graded on OCT	Baseline only (presenting VA study)	Associations of OCT features incl. fibrosis with presenting VA
Channa et al., 2015 (55)	USA (Wilmer Eye Institute)	Retrospective case series	52 eyes	Anti-VEGF (ranibizumab/aflibercept, PRN)	SD-OCT, FA, CF	Atrophy with/without subretinal fibrosis	Mean 33.6 (range 12–73)	BCVA (ETDRS), rate of atrophy/fibrosis
Charles et al., 2023 (40)	France (Lille Catholic Univ.)	Prospective observational	48 eyes (30 reached 5y)	Aflibercept 2 mg, treat-and-extend	OCT, CF	Subretinal fibrosis noted on OCT/fundus	61.7 (mean)	BCVA (ETDRS), injection no., fibrosis/atrophy
Cheung et al., 2019 (41)	Singapore (Asian cohort)	Prospective observational	93 eyes	Anti-VEGF SOC	OCT, FA, ICGA, CFP	Fibrosis from SHRM (reading centre, multimodal)	12	BCVA (logMAR), fibrosis/atrophy incidence
Daniel et al., 2018 (CATT 5-year) (72)	USA multicentre	Prospective cohort	1061	Ranibizumab/bevacizumab, per CATT	CFP, FA, OCT	Fibrotic scar by reading centre (CATT definition)	60	Scar incidence, area, VA, atrophy
Daniel et al., 2019 (13)	USA (CATT multicentre trial)	Prospective cohort within RCT	39 with nonfibrotic scars at year 1 (subset of 1185 total)	Ranibizumab or bevacizumab (monthly, PRN, or mixed)	Colour fundus, FA, OCT	Nonfibrotic scar (flat pigmented lesion without choroidal vessel exposure)	60	Progression to FS, atrophy, VA changes
de la Fuente et al., 2024 (42)	Spain (Navarra + multicentre)	Retrospective ML analysis	36 mo registry	Anti-VEGF real-world	OCT, genetics, clinical data	Fibrosis presence as endpoint	36	Fibrosis/atrophy development (ML prediction)
Dolz-Marco et al., 2017 (22)	USA (NY, UCLA)	Retrospective case series	10	Anti-VEGF (≥3 monthly injections, treat-and-extend or OCT-guided)	CF, FAF, NIR, FA, SD-OCT, OCTA	Type 2 NV progression typically leads to subretinal fibrosis (contrast with regression to Type 1)	≥3 (up to 12)	Regression of Type 2 NV into Type 1, BCVA outcomes, fibrosis prevention
Evans et al., 2020 (34)	USA / UK (multicentre; CATT + IVAN pooled analysis)	Post-hoc analysis of randomized controlled trials	1,731 eyes	Ranibizumab 0.5 mg or Bevacizumab 1.25 mg; monthly vs PRN dosing	SD-OCT	Subretinal fibrosis per CATT/IVAN photographic and OCT reading-centre criteria; evaluated in relation to variability in central retinal (foveal centre-point) thickness	24	BCVA (ETDRS letters), fibrosis and atrophy incidence, association between thickness variability and visual/anatomic outcomes
Finn et al., 2021 (CATT) (56)	USA (multicentre CATT trial)	Cross-sectional imaging analysis within RCT	68	Anti-VEGF (ranibizumab/bevacizumab, CATT protocol)	OCT, CF, FA	Fibrotic scar pixels defined by OCT (subretinal hyperreflective material, PED, MNV, fluids)	24 and 60	Pixel-level associations of MA and FS, lesion evolution
Gianniou et al., 2015 (57)	Switzerland (Lausanne)	Retrospective chart review	76 eyes	Ranibizumab monthly (≥12 mo)	SD-OCT, FA, CF	Fibrosis as end-stage of refractory fluid cases (esp. cystic)	Mean 33.6 (12–73)	BCVA, injection number, fibrosis/atrophy risk
Gillies et al., 2020 (12)	Australia/NZ vs Switzerland	Registry (Fight Retinal Blindness!)	712 (474 ANZ, 321 Swiss)	Anti-VEGF (treat-and-extend vs PRN)	OCT, CF	Subretinal fibrosis noted as cause of VA loss	120	BCVA (logMAR), fibrosis/atrophy, injection no.
Gonzalez-Buendia et al., 2017 (43)	Spain, 7 centres	Observational case series	347 eyes screened; subset with ≥24 mo follow-up	Ranibizumab PRN	OCT, FA, FAF, colour fundus	Presence of minimum area of fibrosis or atrophy (non-foveal)	≥24	BCVA (Snellen→logMAR), OCT morphology, final funduscopic classification (fibrosis/atrophy/preserved)
Gräfe et al., 2020 (52)	Netherlands (Amsterdam UMC)	Imaging diagnostic validation	29 eyes	Previously treated nAMD (mixed)	PS-OCT, FA, ICGA, CFP	Subretinal fibrosis = birefringent collagen fibres (PS-OCT)	Cross-sectional	Diagnostic accuracy, fibrosis detection
Hoffmann et al., 2020 (17)	Germany	Cross-sectional, bilateral nAMD	Not clearly stated; bilateral patients	Anti-VEGF (various)	OCT, FAF, SS-OCTA, microperimetry	Subretinal fibrosis noted as morphologic correlate	Variable, not longitudinal	BCVA, contrast sensitivity (CS), LLVA, reading speed, NEI-VFQ25
Ito et al., 2017 (70)	Japan (Gunma Univ.)	Retrospective	61 eyes (tAMD)	Aflibercept, treat-and-extend	OCT, FA, ICGA	Subfoveal fibrosis (OCT, fundus)	24	BCVA, CMT, injection no., fibrosis incidence
Jackson et al., 2015 (INTREPID) (58)	Multicentre (Europe, 21 sites)	RCT (SRT vs sham + ranibizumab PRN)	230	Ranibizumab PRN ± stereotactic radiotherapy	OCT, FA, CF	Absence/presence of fibrosis as predictor of SRT efficacy	12	Injections needed, BCVA, CST
Jaffe et al., 2019 (CATT 5-year) (30)	USA, multicentre	Secondary analysis of RCT (CATT)	~650 eyes (5-year completers)	Anti-VEGF (ranibizumab/bevacizumab, PRN vs monthly)	OCT, FA, colour photos	Fibrosis reported from treating ophthalmologist + colour photos	60	BCVA (ETDRS letters), lesion size, anatomic features
Janse van Rensburg et al., 2021 (69)	Canada (McGill Univ.)	Retrospective cohort	144 eyes	Anti-VEGF treated nAMD	OCT, FA	Fibrosis underlying ORT (OCT evidence)	5–48 (≥5m)	ORT development, fibrosis presence
Kim et al., 2018 (75)	South Korea (Kim’s Eye Hosp.)	Retrospective	293 PCV eyes	Ranibizumab or aflibercept PRN	OCT, FA, ICGA	Fibrotic scar at 12m	12	BCVA, fibrosis incidence
Kim et al., 2020 (45)	South Korea (Kim’s Eye Hosp.)	Retrospective	195 type 3 MNV eyes	Anti-VEGF (varied regimens)	OCT, FA, ICGA	Fovea-involving fibrotic scar vs GA	47.5 (mean)	BCVA, GA vs scar vs none
Kim et al., 2022 (44)	South Korea (Yeungnam Univ.)	Retrospective	68 eyes (m-PED with prechoroidal cleft)	Anti-VEGF SOC	OCT, FA, ICGA	Fibrotic scar from hyperreflective band (layer 2)	12	BCVA, fibrosis incidence, PED changes
Le et al., 2021 (71)	France (Créteil, multicentre)	Retrospective case series	29	Anti-VEGF (naïve)	SD-OCT	Progression type 2 MNV → fibrovascular PED (fibrosis surrogate)	44 (mean 3.7y)	BCVA, anatomical change
Lenhof et al., 2025 (25)	France (6 centres)	Multicentre retrospective	420	Anti-VEGF (naïve, PRN/TAE)	SD-OCT, FA	Subretinal fibrosis (standardized def.)	36	Cumulative incidence, BCVA
Lindenberg et al., 2025 (46)	Canada	Post-hoc analysis of SEVEN-UP trial	Small sample (exact not clear)	Long-term anti-VEGF	OCT, colour fundus	Fibrosis vs atrophy defined by SHRM thickness/reflectivity on OCT and colour photos	~84+	SHRM thickness, reflectivity, fibrosis/atrophy classification
Liu et al., 2024 (59)	China	Retrospective cohort	64 eyes, 64 patients	Anti-VEGF (various, median 4 inj/yr)	OCT, OCTA, FA, multimodal	SF defined as dense SHRM on OCT with RPE/EZ/ELM loss; confirmed by FA staining	12	BCVA (logMAR), OCTA vessel parameters, fibrosis risk
Llorente-González et al., 2022 (60)	Spain (17 centres)	Ambispective multicentre	359	Anti-VEGF per Spanish guidelines	OCT, FA	Fibrosis (clinical/OCT)	36	BCVA, fibrosis/atrophy incidence, associated with fluid
Maruyama-Inoue et al., 2020 (47)	Japan	Retrospective	Not clear (nAMD cohort)	Anti-VEGF	OCT, FA	Subretinal fibrosis (CF/OCT definition)	Variable	BCVA
Miere et al., 2015 (23)	France (Créteil)	Prospective, single-centre	49 eyes, 47 patients	Anti-VEGF (mean 13 inj; some naïve)	OCT, FA, OCTA, fundus	FA: staining with minimal leakage; OCT: >50% compact hyperreflective sub-RPE lesion ≥100 µm thick	Mean ~44 months disease duration	BCVA (ETDRS logMAR), OCTA vascular patterns
Motschi et al., 2021 (61)	Austria	Prospective diagnostic imaging	57 eyes	Anti-VEGF (ongoing)	PS-OCT, CFP	Birefringence-based automated segmentation (optic axis uniformity, birefringence signal)	Cross-sectional	Fibrotic lesion area quantification, agreement with CFP
Ohayon et al., 2020 (62)	France (Créteil)	Retrospective	30 eyes	Single anti-VEGF injection	OCT, FA, ICGA	Multilayered fibrovascular PED: hyperreflective band (layer 2) considered fibrotic	1 month	OCT layer thickness, BCVA (ETDRS)
Okeagu et al., 2021 (AREDS2) (48)	USA (AREDS2 sites)	Prospective cohort	594 eyes with incident nAMD	Anti-VEGF (clinical practice, self-reported)	Colour fundus photography (central grading)	Subretinal fibrosis on CFP as principal cause of poor vision	24	BCVA (ETDRS)
Papavasileiou et al., 2015 (63)	UK (multi-centre chart review)	Retrospective, non-comparative	26 eyes	Aflibercept fixed (q8w after 3 loading)	SD-OCT, FA	Presence of subretinal fibrosis noted on OCT and fundus	14	BCVA (logMAR), CRT
Querques et al., 2020 (18)	Italy (Milan, Rome)	Prospective, cross-sectional	41 eyes (remission)	Previously treated anti-VEGF, ≥6 mo off	Multimodal: MC imaging, OCT, FAF, OCTA, microperimetry	Fibrocellular vs fibrovascular phenotype based on MC imaging	Cross-sectional	BCVA (logMAR), microperimetry
Ramtohul et al., 2022 (67)	Multinational (France, Italy, USA)	Retrospective, longitudinal	30 eyes	Anti-VEGF (ranibizumab, aflibercept, bevacizumab); treat-extend / PRN	OCT, OCTA, FA, FAF, CF	Subretinal fibrosis (CATT definition: yellow lesion on CF + hyperreflective scar OCT)	Mean ~50 (up to 123)	BCVA (logMAR), fibrosis incidence, Cox regression
Roberts et al., 2016 (15)	Austria (Vienna)	Case series, imaging validation	15 eyes	Previously treated nAMD, advanced stage	PS-OCT, SD-OCT, FA, CF	Subretinal fibrosis identified by PS-OCT birefringence vs conventional imaging	Cross-sectional	BCVA, PS-OCT segmentation
Roberts et al., 2021 (24)	Austria (Vienna)	Prospective, cross-sectional	60 eyes	≥12 mo anti-VEGF (mixed)	PS-OCT, SS-OCTA, SD-OCT, CF	SF defined by CF + OCT, confirmed by PS-OCT	Cross-sectional	BCVA (ETDRS), OCTA vessel metrics
Roberts et al., 2022 (31)	Austria (Vienna)	Prospective, longitudinal	45 eyes (treatment-naïve)	Aflibercept treat-and-extend	SD-OCT, CF, FA, PS-OCT, OCTA	SF defined by CF (yellow scar), FA (early hypo/late stain), PS-OCT birefringence	12	BCVA (ETDRS), OCTA vessel metrics, SF incidence
Roberts et al., 2019 (32)	Austria (Medical Univ. Vienna)	Prospective observational	50	Anti-VEGF (3 loading injections)	PS-OCT, SD-OCT, FA	Subretinal fibrosis detected by birefringence on PS-OCT	3	SHRM volume, fibrosis detection, BCVA
Romano et al., 2023 (8)	Italy, Singapore	Retrospective, multicentre	225 eyes, 10-year follow-up	Anti-VEGF (PRN, varied)	OCT, FA, CF, OCTA	Fibrosis graded as sub-RPE, subretinal, mixed	120	BCVA (ETDRS), fibrosis incidence, risk factors
Romano et al., 2022 (73)	Italy (Milan)	Cross-sectional, single-centre	48	Anti-VEGF treated eyes with RPE tears	Multimodal (OCT, FAF, NIR-AF, microperimetry)	Fibrosis as repair tissue after RPE tear	≥12	BCVA, retinal sensitivity (MP), RPE resurfacing, AF recovery
Schranz et al., 2024 (21)	Austria (Vienna)	Cross-sectional observational	30	≥1-year anti-VEGF	CFP, SD-OCT, PS-OCT, MP	Retinal fibrosis defined by CFP/OCT/PS-OCT	≥12	Retinal sensitivity (MP), multimodal imaging correlation
Souied et al., 2020 (9)	France (Créteil)	Retrospective	91 (44 cross-sectional, 47 longitudinal)	Long-term anti-VEGF (as needed)	SD-OCT, FA, CFP	Fibrotic lesions (>50% compact hyperreflective material)	Up to >60 (5+ years)	Lesion classification, fibrosis progression pathways
Tan et al., 2024 (64)	Singapore	Cross-sectional	87 AMD patients (140 eyes), 66 controls	Not treatment-specific	Multimodal: OCT, OCTA, IR, CFP, microperimetry	End-stage fibrosis defined on OCT/CFP	Cross-sectional	Microperimetry retinal sensitivity (dB), fixation stability
Teo et al., 2020 (FRB!) (49)	Multinational registry (AU, NZ, SG, CH)	Longitudinal registry	2,914 eyes	Anti-VEGF	CFP, OCT, FA	Physician-graded subretinal fibrosis (white/yellow mound, staining on FA)	Up to 120 (10 yrs)	BCVA (letters), fibrosis incidence/prevalence
Teo et al., 2024 (50)	Singapore	Case-control (Phenotyping Asian Macular Diseases)	356 eyes	Anti-VEGF	OCT, CFP, FA	Subfoveal fibrosis: hyperreflective material (OCT) + fundus pallor/FA staining	12	BCVA (letters)
Toth et al., 2019 (33)	USA (CATT trial centres)	Cross-sectional analysis within RCT	68 (subset of 1185)	Ranibizumab/bevacizumab (CATT protocol)	SD-OCT, CFP, FA	Fibrotic scar (CATT Reading Centre)	24	OCT lesion features, subretinal thickness, VA
Willoughby et al., 2015 (16)	USA (CATT multicentre)	Prospective cohort (within RCT)	1185	Ranibizumab/bevacizumab, monthly or PRN	TD-OCT, SD-OCT, CFP, FA	SHRM persistence linked to fibrosis	24	SHRM location/dimensions, VA, fibrosis/GA
Wu et al., 2022 (65)	China (Shanghai)	Retrospective cohort	20	Aflibercept or ranibizumab (≥3 monthly)	OCTA, OCT	Subretinal fibrosis complicating NVAMD	12	CNV remodelling, fibrosis progression, BCVA
Yu et al., 2023 (AVENUE post-hoc) (51)	USA, multicentre (RCT reanalysis)	Post-hoc of AVENUE trial	207 eyes	Faricimab / ranibizumab	OCT	SHRM with boundary remodelling vs persistent HRM (fibrosis-related phenotype)	9	BCVA (letters), atrophy development
Zhao et al., 2021 (66)	China (Beijing, PUMCH)	Retrospective	244 patients (nAMD 155 eyes, PCV 89 eyes)	Anti-VEGF (varied; delays due to COVID-19)	OCT, OCTA, FA, ICGA	Sub-macular scar/fibrosis formation	~8	BCVA, fibrosis incidence, impact of delayed therapy

Summary of study design, sample size, treatment regimens, imaging modalities, fibrosis definitions, follow-up duration, and outcome measures across included studies.

AF, Autofluorescence; AMD, Age-related macular degeneration; ANZ, Australia and New Zealand; BCVA, Best-corrected visual acuity; BR, Boundary remodelling; CATT, Comparison of Age-related Macular Degeneration Treatments Trials; CF, Colour fundus; CFP, Colour fundus photography; CH, Switzerland; CNV, Choroidal neovascularization; MNV, Macular neovascularization; CMT, Central macular thickness; CST, Central subfield thickness; CRT, Central retinal thickness; CS, Contrast sensitivity; dB, Decibels (microperimetry sensitivity); ELM, External limiting membrane; ETDRS, Early Treatment Diabetic Retinopathy Study; FA, Fluorescein angiography; FAF, Fundus autofluorescence; FRB!, Fight Retinal Blindness! registry; FS, Fibrotic scar; GA, Geographic atrophy; HRF, Hyperreflective foci; HRM, Hyperreflective material; ICGA, Indocyanine green angiography; ICG, Indocyanine green; inj, Injections; IR, Infrared reflectance; IRF, Intraretinal fluid; SRF, Subretinal fluid; IVAN, Inhibition of VEGF in Age-related choroidal Neovascularisation trial; LLVA, Low luminance visual acuity; logMAR, Logarithm of the minimum angle of resolution; MA, Macular atrophy; MC imaging, Multicolour imaging; ML, Machine learning; MP, Microperimetry; N/A, Not applicable; NIR, Near-infrared reflectance; NIR-AF, Near-infrared autofluorescence; NV, Neovascular; NVAMD, Neovascular age-related macular degeneration; OCT, Optical coherence tomography; SD-OCT, Spectral-domain OCT; SS-OCTA, Swept-source OCT angiography; OCTA, OCT angiography; ORT, Outer retinal tubulation; PCV, Polypoidal choroidal vasculopathy; PED, Pigment epithelial detachment; PRN, Pro re nata (as needed); PROs, Patient-reported outcomes; PS-OCT, Polarization-sensitive OCT; q2w, Every 2 weeks; q4w, Every 4 weeks; q8w, Every 8 weeks; RCT, Randomized controlled trial; RPE, Retinal pigment epithelium; SD, Standard deviation; SF, Subretinal fibrosis; SRFi, Subretinal fibrosis eyes; SHRM, Subretinal hyperreflective material; SRT, Stereotactic radiotherapy; TAE, Treat-and-extend; tAMD, Typical AMD; TD-OCT, Time-domain OCT; UK, United Kingdom; USA, United States of America; VA, Visual acuity.

### 3.2 Imaging modalities and fibrosis definitions

Definitions of fibrosis varied widely across studies, encompassing OCT-based dense subretinal hyperreflective material (SHRM), CFP-defined fibrotic scar, PS-OCT birefringence, and multimodal criteria (**Supplementary Tables S2**, **S3**). OCT was most frequently employed (n = 25), typically defining fibrosis as the transition of SHRM that evolves into a dense, homogeneous, hyperreflective scar, often accompanied by RPE disruption or elevation (16, 25, 41, 50, 59).

In contrast, CFP-based studies (n = 18) defined fibrosis according to the CATT and IVAN trial criteria, namely, well-demarcated white or yellow subretinal plaques showing late hyperfluorescent staining and corresponding loss of retinal detail on FA, thereby distinguishing fibrotic scars from nonfibrotic scars of atrophy (8, 19, 48, 72). FAF (n=4).

FAF studies (n=4) described regions of hypo- or hyperautofluorescence aligning with fibrotic tissue (43, 73). Polarisation-sensitive OCT (n=5) uniquely detected birefringent collagen, enabling automated volumetric quantification of fibrotic tissue (15, 24, 31, 32, 62, 74).

A few OCT-A studies (n=3) evaluated flow characteristics within or adjacent to fibrotic plaques, such as signal voids, vascular pruning, or network rarefaction, rather than directly defining fibrosis.

**Figure 2:**
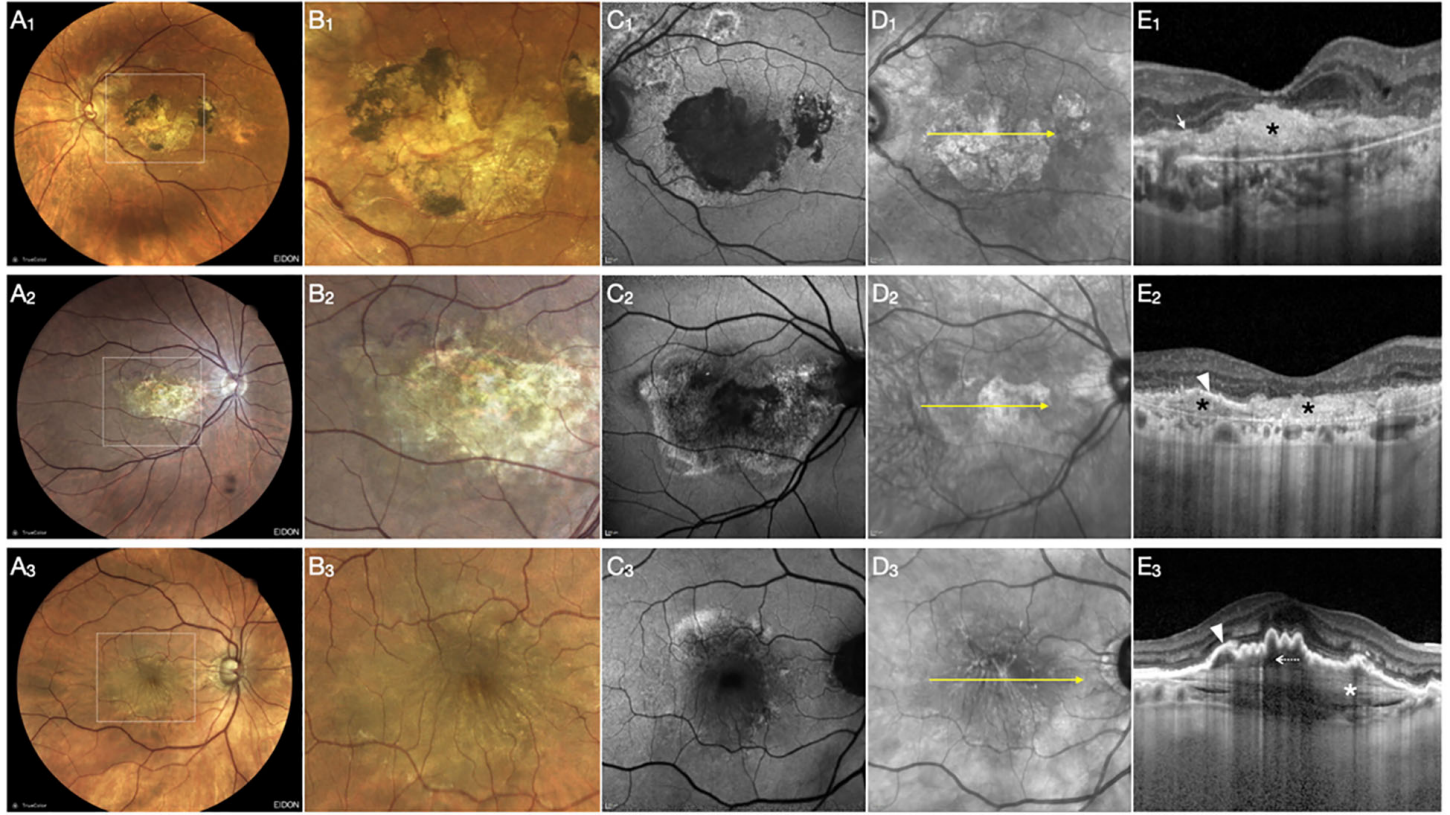
Multimodal imaging findings in fibrosis secondary to neovascular AMD, categorized according to the anatomical location of fibrotic tissue. The first row **(A1–E1)** presents pure subretinal fibrosis, the second row **(A2–E2)** a mixed type, and the third row **(A3–E3)** sub-RPE fibrosis. Color fundus photography **(A1–A3)** with corresponding magnified views **(B1–B3)** provides clinical visualization of the fibrotic areas. Fundus autofluorescence (FAF) imaging **(C1–C3)** demonstrates distinct patterns. In pure subretinal fibrosis **(C1)**, a sharply demarcated hypoautofluorescent area corresponds to complete RPE loss. In the mixed type **(C2)**, a smaller hypoautofluorescent area is surrounded by mottled FAF signal, indicating partial RPE preservation. In sub-RPE fibrosis **(C3)**, the FAF signal is relatively preserved without a sharply demarcated hypoautofluorescent defect. Near-infrared reflectance (NIR) imaging **(D1–D3)** demonstrates corresponding reflectance alterations overlying the fibrotic lesions (yellow arrows). Optical coherence tomography (OCT) B-scans **(E1–E3)** demonstrate three distinct structural patterns. In pure subretinal fibrosis **(E1)**, fibrotic tissue (black asterisk) lies entirely beneath the neurosensory retina, with complete loss of the RPE and photoreceptor layers (white arrow). In the mixed type **(E2)**, partial preservation of the RPE is observed temporally (white arrowhead), and fibrotic tissue is located both beneath and within the retinal layers (black asterisks). In sub-RPE fibrosis **(E3)**, fibrotic tissue is confined between the macular neovascular complex (dotted white arrow) and Bruch’s membrane.

Approximately 10 studies employed multimodal imaging, combining OCT, FA, FAF, and CFP, to improve the identification and longitudinal tracking of fibrotic changes. Representative examples of these multimodal features are illustrated in **Figure 2**, demonstrating the characteristic appearance of fibrosis across imaging modalities. Interestingly, Le et al. reported that type 2 MNV can evolve into a fibrovascular pigment epithelial detachment within the first 3 months of anti-VEGF therapy, representing a distinct morphological pathway toward sub-RPE fibrosis that may nevertheless preserve good visual acuity (71). These imaging-derived definitions represent related but distinct phenotypes along a fibrotic spectrum and should not be considered interchangeable. Studies assessing inter-rater reliability and reproducibility of fibrosis grading across imaging modalities are summarized in **Supplementary Table S4**.

### 3.3 Quantification methods

Fibrosis, strictly defined, is a histopathological process characterized by collagen deposition and fibrocellular tissue formation beneath the neurosensory retina. Imaging studies, therefore, rely on structural surrogates rather than true histologic confirmation.

Most studies applied categorical grading of fibrosis (present vs. absent; fibrotic vs nonfibrotic) (72). On CFP, the earliest and most widely adopted definitions, originating from the CATT and IVAN trials, classified fibrotic scars as well as demarcated white or yellow subretinal plaques with late fluorescein staining, distinguishing them from nonfibrotic scars or atrophic lesions (14, 19, 72).

OCT-based assessments subsequently linked these CFP features to structural correlates. Most studies described subretinal fibrosis as dense SHRM on or above the RPE, and sub-RPE fibrosis as hyperreflective material beneath an elevated or disrupted RPE band, often corresponding to fibrovascular PED. However, not all sub-RPE fibrovascular tissue manifests as a “fibrotic scar” on CFP, highlighting the partial overlap between imaging modalities.

Manual area or thickness measurements of SHRM or scar tissue were occasionally reported on B-scan or *en face* OCT images. At the same time, PS-OCT uniquely enabled volumetric birefringent-based quantification of collagen (15, 32, 61). Two recent studies trialled AI-based segmentation of OCT images (42, 59), though neither included external or histologic validation.

### 3.4 Incidence and time course of fibrosis

The reported incidence of macular fibrosis varied substantially by MNV type, treatment regimen, and duration of follow-up. Subgroup analyses by lesion subtype and treatment regimen are presented in **Supplementary Table S5**. Across 11 studies, the pooled cumulative incidence was 29.4% (95% CI, 25.1–34.1), with marked heterogeneity (I² = 100%). The incidence increased further in long-term cohorts, reaching 40-50% by 5 years of continuous anti-VEGF therapy. When stratified by MNV type, the risk was lowest in PCV (7.5%, 95% CI 3.7–14.5) (41, 75) and highest in type 2 MNV (61.5%, 95% CI 22.4–89.8) (8, 9). Type 1 lesions demonstrated a comparatively low incidence (15.1%, 95% CI 14.9–15.2), while type 3 lesions showed intermediate rates (29.8%, 95% CI 29.4–30.3) (25). Mixed lesions had a pooled incidence of 41.0% (95% CI 36.4–45.7). Regimen-related differences were also evident: the pooled incidence was 40.0% (95% CI, 39.9–40.1) with monthly dosing, 46.6% (95% CI, 45.0–48.2) under *pro re nata* regimens, and 24.2% (95% CI, 15.8–35.1) with treat-and-extend. Longitudinal analyses demonstrated a steady increase in the cumulative incidence of fibrosis over time, with rates approaching 40–50% at five years (CATT, Gillies 2020) (12, 72) and nearly two-thirds of eyes affected at ten years in Romano et al. (8).

### 3.5 Functional outcomes

#### 3.5.1 Best corrected visual acuity

BCVA was consistently worse in eyes that developed fibrosis. A pooled random-effects meta-analysis of six studies comprising more than 3,500 eyes demonstrated a mean BCVA deficit of 29.3 ETDRS letters (95% CI −47.1 to −11.5; I² = 96.8) in fibrotic compared with nonfibrotic eyes (8, 25, 36, 48, 50, 72). This effect was apparent across all follow-up intervals. At 12–24 months, eyes with fibrosis typically had 10–20 fewer letters than controls, as shown in CATT, IVAN, and registry studies (16, 19, 72). At 5 years, CATT data revealed a mean BCVA of 48 letters in eyes with fibrotic scars, compared with 73 letters in eyes with nonfibrotic scars and 62 letters in eyes without scarring (8). Long-term outcomes were similarly poor: Romano et al. (8) reported a mean BCVA of 44 ± 22 letters in fibrotic eyes versus 63 ± 17 in nonfibrotic eyes at ten years. Study-level visual acuity outcomes and follow-up durations are detailed in **Supplementary Table S6**.

Interpretation of pooled BCVA differences is limited by heterogeneity in visual acuity measurement scales, variable follow-up intervals, inconsistent baseline visual acuity adjustment, and incomplete reporting of cataract status or cataract surgery. In eyes with advanced disease, floor effects may further attenuate the detectability of changes in visual acuity.

#### 3.5.2 Other functional outcomes

Evidence for functional outcomes beyond BCVA was more limited but consistently unfavourable for eyes with fibrosis (**Supplementary Table S7**). Microperimetry demonstrated marked reductions in mesopic retinal sensitivity, typically in the range of 8–15 dB, compared with preserved retinal areas, as reported by Querques (2020), Schranz (2024), and Tan (2024) (18, 21, 64). Contrast sensitivity (CS), measured with the Pelli-Robson chart, was significantly reduced in fibrotic eyes compared with nonfibrotic eyes. CS loss correlated strongly with declines in vision-related quality of life (NEI-VFQ-25) scores (17).

The same study also showed worse low-luminance visual acuity (LLVA) under a 2.0-log ND filter and slower reading speed, although the latter was not stratified by fibrosis status. Collectively, these findings suggest that subretinal fibrosis adversely affects not only high-contrast acuity but also contrast sensitivity, mesopic retinal sensitivity, and patient-perceived visual function.

### 3.6 Factors associated with fibrosis

**Figure 3:**
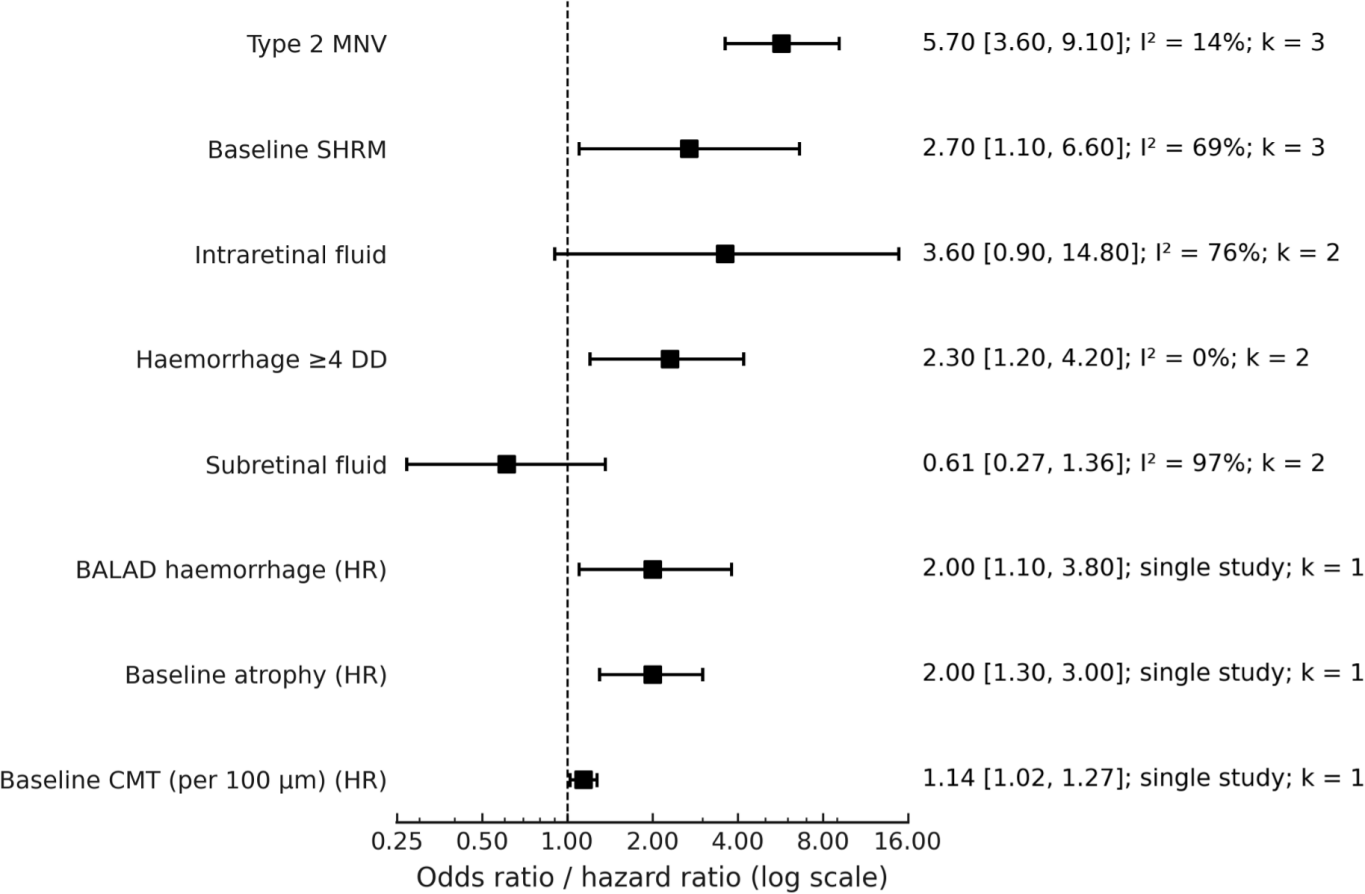
Forest plot of baseline associations of subretinal fibrosis. Odds ratios (ORs) are shown on a log scale with 95% confidence intervals (CIs). Baseline associations with incident fibrosis (red) include Type 2 MNV (OR 5.7, 95% CI 3.6–9.1; I² = 14%; k = 3), baseline SHRM (OR 2.7, 95% CI 1.1–6.6; I² = 69%; k = 3), intraretinal fluid (OR 3.6, 95% CI 0.9–14.8; I² = 76%; k = 2), and haemorrhage ≥4 DD (OR 2.3, 95% CI 1.2–4.2; I² = 0%; k = 2). Protective associations (blue) include subretinal fluid (OR 0.61, 95% CI 0.27–1.36; I² = 97%; k = 2). Additional associations reported in single studies included BALAD haemorrhage (HR 2.0, 95% CI 1.1–3.8; k = 1), baseline atrophy (HR 2.0, 95% CI 1.3–3.0; k = 1), and baseline central macular thickness (HR 1.14, 95% CI 1.02–1.27; k = 1). The dashed vertical line indicates the null effect (OR = 1).

Several imaging and clinical features were consistently associated with the development of fibrosis across included studies (**Figure 3**, **Table 2**). The most consistently associated imaging and clinical features were the presence of type 2 MNV (OR 5.7, 95% CI 3.6–9.1), baseline SHRM (OR 2.7, 95% CI 1.1–6.6), and intraretinal fluid (OR 3.6, 95% CI 0.9–14.8). Large baseline haemorrhage (≥ 4-disc diameters) was also associated with a higher risk (OR 2.3, 95% CI 1.2–4.2). By contrast, subretinal fluid demonstrated a pooled odds ratio of 0.61 (95% CI 0.27–1.36), with substantial heterogeneity, compatibles with no clear association with fibrosis across studies.

**Table 2:** Imaging and clinical factors associated with Fibrosis in nAMD.

Imaging / Clinical feature	Timing / Definition used across studies	Representative studies	Summary of association with fibrosis
Type 2 macular neovascularization (MNV)	Baseline lesion subtype	Romano 2023 (8); Souied 2020 (9); Lenhof 2025 (25); Liu 2024 (59)	Consistently associated with higher odds of fibrosis across cohorts and registries
Subretinal hyperreflective material (SHRM)	Baseline presence or persistence on OCT	Willoughby 2015 (16); Casalino 2018 (35); Kim 2018 (75); Ramtohul 2022 (67); Liu 2024 (59)	Strongly and consistently associated with fibrosis across imaging definitions
Intraretinal fluid (IRF)	Presence or persistence during follow-up (time-updated)	PRECISE (Chandra 2024) (68); Roberts 2022 (31); Lenhof 2025 (25); Llorente-González 2022 (60)	Associated with fibrosis in several studies; pooled estimates show substantial heterogeneity
Subretinal fluid (SRF)	Baseline or during follow-up	Gianniou 2015;57 Llorente-González 2022 (60); PRECISE (Chandra 2024) (68)	Pooled estimate compatible with no clear association with fibrosis (OR 0.61, 95% CI 0.27–1.36; high heterogeneity)
Large subretinal haemorrhage (≥4 disc diameters)	Baseline haemorrhage extent	Daniel 2018 (CATT) (13); Ramtohul 2022 (67)	Associated with increased odds of subsequent fibrosis
Bacillary layer detachment (BALAD)	Baseline OCT finding	Ramtohul 2022 (67)	Associated with higher fibrosis risk in single-study analyses
Baseline visual acuity	ETDRS letters at presentation	PRECISE (Chandra 2024) (68); Romano 2023 (8); Angermann 2022 (36)	Lower baseline visual acuity frequently observed in eyes with fibrotic features at presentation
Baseline central macular thickness (CMT)	Per 100-µm increase	Daniel 2018 (CATT) (13)	Modestly associated with fibrosis in single-study analyses
Baseline atrophy	Presence of geographic or outer retinal atrophy	Daniel 2018 (CATT) (13)	Associated with fibrosis in single-study analyses

MNV, Macular neovascularization; SHRM, Subretinal hyperreflective material; IRF, Intraretinal fluid; SRF, Subretinal fluid; OR, Odds ratio; HR, Hazard ratio; aHR, Adjusted hazard ratio; VA, Visual acuity; DD, Disc diameter; CNV, Choroidal neovascularization; RAP, Retinal angiomatous proliferation; PCV, Polypoidal choroidal vasculopathy; BALAD, Bacillary layer detachment; RPE, Retinal pigment epithelium; ETDRS, Early Treatment Diabetic Retinopathy Study; EZ, Ellipsoid zone; ELM, External limiting membrane; ORT, Outer retinal tubulation; ML, Machine learning; GEE, Generalized estimating equation; GLM, General linear model.

Additional single-study baseline associations included bacillary layer detachment, baseline atrophy, and increased or fluctuating central subfield thickness (CST). In particular, Evans et al. reported that greater CST variability over serial visits was independently associated with subsequent fibrotic or atrophic scarring, even after adjustment for baseline lesion type and treatment intensity (34).

Poor baseline visual acuity and lesion subtype were consistently associated with fibrosis. In a Japanese aflibercept treat-and-extend cohort, Ito et al. found subfoveal fibrosis in 23% of eyes, occurring more frequently in classic than occult CNV (OR 7.6, p < 0.01) (70). Similarly, the PRECISE registry (68) identified fibrotic or fibro-vascular components at baseline in approximately 17% of treatment-naïve eyes, with low baseline vision (< 54 letters) representing the strongest independently associated covariate (68). Janse van Rensburg et al. further reported that Type 2 MNV and the presence of subretinal fibrotic tissue was strongly associated with outer retinal tubulation formation (OR 22.2 and 3.1, respectively), supporting an association between subretinal fibrosis and structural retinal remodelling (69).

### 3.7 Risk of bias

Risk of bias assessment using the QUIPS tool is summarised in **Supplementary Table S8**. Secondary analyses of large randomized controlled trials were generally rated as low risk across most domains (19, 72). Registry-based studies were typically judged to be at moderate risk, most often due to incomplete outcome ascertainment and variable follow-up (12, 49, 60). Smaller retrospective cohorts, particularly those including fewer than 50 eyes, tended to be at higher risk of bias in the domains of attrition, outcome measurement, and statistical adjustment (45, 55, 62). These risks of bias informed the GRADE assessments and underpin the low to very low certainty ratings assigned to most associations. Certainty of evidence ratings according to GRADE are presented in **Supplementary Tables S9** and **S10**.

## 4 Discussion

### 4.1 Main findings in the context of existing literature and implications for research

This systematic review of 58 studies confirms that macular fibrosis is a common and significant sequela of nAMD undergoing anti-angiogenic therapy. Within the first 12–24 months of treatment, fibrosis develops in approximately 10–25% of eyes receiving fixed monthly dosing, 20–25% of eyes on *PRN* (reactive) dosing, and 15-20% under treat-and-extend (T&E) protocols (13, 19).Long-term data indicate that up to 40–50% of treated eyes exhibit fibrosis by 5 years (8, 12, 50, 76, 77). Taken together, the evidence suggests that subretinal fibrosis is the rule rather than the exception in late-stage nAMD. Our review identified several independent covariates of fibrosis that emerged consistently.

Importantly, pooled estimates in this review should be interpreted as signals of association rather than definitive or interchangeable effect sizes. Given the substantial heterogeneity in fibrosis definitions, imaging modalities, and study designs, these analyses were not intended to imply biological equivalence across imaging phenotypes, but rather to identify consistent directional associations across the existing literature.

Among imaging features, persistent SHRM on OCT showed the most consistent associated with subsequent fibrosis across studies (16, 24, 32). Other factors associated with fibrosis included the presence of IRF during follow-up, poor baseline visual acuity, and type 2 MNV. Findings from the PRECISE study similarly indicate that eyes with poorer baseline VA more frequently exhibit fibrotic features at presentation, highlighting the importance of early recognition of fibrotic phenotypes rather than establishing definitive prognostic relationships (68, 74).

According to GRADE criteria adapted for prognostic research, the certainty of evidence supporting most associations identified in this review was rated as low or very low. These ratings primarily reflect inconsistency across studies, indirectness due to heterogeneous imaging definitions, and residual confounding inherent to observational designs.

Evidence for submacular haemorrhages (SMH) as a risk factor is more limited but supportive. Large baseline or recurrent haemorrhages, particularly those ≥4-disc diameters, were associated with higher odds of fibrosis in CATT, consistent with the concept that subretinal blood promotes fibrocellular organization through iron-induced RPE and photoreceptor injury (67, 72). While SMH has not been uniformly quantified across studies, the available data suggest a moderate independent association with fibrotic scarring.

In contrast, SRF was often protective, correlating with lower fibrosis rates, possibly because it represents a spatial separation between the neurosensory retina and the neovascular complex, delaying fibrotic organization (57, 60). However, SRF occurs more frequently in type 1 (occult) MNV, which itself carries a lower intrinsic risk of fibrotic scar formation; therefore, the apparent protective effect of SRF may partly reflect lesion-type confounding rather than a direct anti-fibrotic influence.

Cheong et al. conducted a recent systematic review and meta-analysis that reported similar fibrosis incidence rates and identified many of the same risk and protective factors for scarring (76). Our analysis builds on this foundation by adding treatment regimen-specific insights: we observed that eyes managed with PRN anti-angiogenic treatment had higher one- and two-year fibrosis rates than those under continuous monthly or treat-and-extend therapy, potentially reflecting the benefit of more sustained angiogenic suppression (13, 19). Although proactive regimens (fixed monthly or treat-and-extend) are associated with lower cumulative fibrosis at 12–24 months compared with reactive PRN dosing, consistent with a delay in fibrosis onset, the “fibrosis gap” narrows by approximately 5 years, with a substantial proportion of eyes developing fibrosis even under treat-and-extend protocols. Notably, both our review and Cheong et al. observed that most fibrotic scarring emerges within the first year of treatment, suggesting an early transition toward fibrocellular remodelling in a subset of eyes (‘angio–fibrotic transition’ concept), which current anti-VEGF therapy may not fully prevent (76). However, several studies suggest that fibrosis tends to appear early, predominantly in eyes that are already severely affected at baseline, either due to delayed initiation of anti-VEGF therapy or inherently aggressive disease activity, rather than representing *de novo* early scarring in otherwise mild cases (54, 69).

Emerging evidence suggests that maintaining steady disease control, with minimal lapses or recurrences, may be more critical than the exact extension interval used in treat-and-extend protocols. For example, a 10-year follow-up by Romano et al. identified large fluctuations in central retinal thickness as one of the strongest associations of new fibrosis; eyes with frequent recurrences had substantially higher rates of scarring (8). Conversely, the ALTAIR trial demonstrated comparable visual and anatomic outcomes between 2-week and 4-week extension increments over 2 years, though fibrotic scarring was not a dedicated endpoint in this analysis (78).

Limited phenotypic data suggest that proactive regimens may primarily delay or reduce subretinal fibrocellular scarring. At the same time, sub-RPE fibrosis likely represents a natural progression of neovascular lesions and may persist despite optimal exudation control. Even monthly anti-angiogenic regimens do not eliminate the risk of fibrosis (79). Thus, controlling or preventing fibrosis remains an unmet need in current practice.

The genetic susceptibility to subretinal fibrosis in nAMD remains unclear. While experimental and translational work has implicated profibrotic signalling pathways such as TGF-β, PDGF, Wnt, and inflammatory mediators such as TNF-α and matrix metalloproteinases in myofibroblast recruitment and collagen deposition beneath the neurosensory retina, no included clinical study demonstrated a reproducible association between germline variants (e.g., CFH, ARMS2/HTRA1, TGF-β1 polymorphisms) and the subsequent development of imaging-defined fibrosis under anti-VEGF therapy. Instead, fibrosis risk in real-world and trial cohorts was driven predominantly by lesion phenotype (notably type 2 MNV), baseline or persistent SHRM, intraretinal fluid, large subretinal haemorrhage, and unstable anatomical control over time (8, 12, 16, 25, 34, 49, 50). Aqueous humour cytokine profiling studies have shown increased levels of TGF-β1, IL-6, and VEGF in eyes with established fibrosis, consistent with an active wound-healing microenvironment (37). However, these reflect local inflammatory milieu rather than inherited susceptibility. Pre-clinical work has further characterized profibrotic cascades in experimental models (11, 80), and reviews of anti-fibrotic targets have highlighted these same molecular pathways as potential therapeutic avenues (79, 81). Taken together, current evidence supports the biological plausibility of targeted anti-fibrotic pathways, but no clinically actionable genetic signature for fibrosis has yet been identified.

It is well established that fibrotic scarring in nAMD is associated with poorer visual acuity outcomes, and our findings reaffirm this link. On average, BCVA of eyes that developed fibrosis had 25–30 fewer ETDRS letters by 3–5 years of treatment compared to nonfibrotic eyes, and our meta-analysis found a similar 29-letter deficit (25, 36, 77). This gap emerges early (already 15–20 letters by 1 and 2 years) and tends to widen over time. This degree of vision loss underscores the severe functional impact of fibrotic scarring. Our analyses extend beyond BCVA by considering other functional metrics. Although only sparingly reported to date, they demonstrate that fibrosis profoundly impairs broader visual function: microperimetry studies demonstrate 8–15 dB reductions in retinal sensitivity over fibrotic areas; LLVA and CS are significantly impaired in eyes with scars; and one study noted consistently lower vision-related quality of life (NEI VFQ-25 scores) in patients with fibrotic scarring (17, 18, 21, 64, 82).

While BCVA consistently worsened in eyes with fibrosis, BCVA alone does not fully capture the functional impact of fibrotic scarring. Floor effects in advanced disease, cataract progression or surgery, and competing atrophic changes may partially confound observed differences in visual acuity, particularly at longer follow-up intervals.

Future studies should incorporate additional functional assessments to evaluate retinal sensitivity in eyes with fibrotic scars. Early microperimetry data indicate localized sensitivity loss corresponding to areas of fibrosis; correlating such functional maps with detailed structural characteristics of fibrosis (e.g., scar area, thickness, location, and vascularity) can identify which aspects of scarring are most detrimental to vision, inform clinical management, and help design therapies aimed at preserving functional vision despite fibrotic changes. In addition, emerging metrics such as the quantitative contrast sensitivity function (qCSF), which have recently been integrated into the MACUSTAR protocol and other AMD and diabetic retinopathy trials (83), offer a more granular assessment of spatial-frequency-dependent vision loss and could complement microperimetry in capturing subtle functional deficits associated with fibrosis.

Across pooled analyses, heterogeneity was extreme, with I² values ranging from 96% to 100%, reflecting marked variability in fibrosis definitions, imaging modalities, and study designs. Extreme heterogeneity across studies reflects not a failure of methodology, but a fundamental absence of standardized, operative definitions of fibrosis in neovascular age-related macular degeneration. Importantly, our review also revealed heterogeneity in how fibrosis is defined and measured, representing a critical barrier to progress. Imaging criteria varied across studies; for instance, fibrosis was first defined as a well-demarcated, whitish subretinal lesion on CPF or as the presence of late-phase staining on FA, whereas others relied on OCT features, such as dense subretinal hyperreflective scar tissue disrupting the RPE (16, 19, 22, 32, 84). The majority of studies used binary classification (fibrosis present/absent) or qualitative grading, with only a few attempting to quantify fibrotic lesion size or volume (15, 21, 85). None of these approaches has yet been validated against histopathology or used in multicenter trials. In short, the current literature employs a patchwork of definitions and grading scales for fibrosis, hindering direct comparisons of outcomes across studies. This is also reflected in recent studies, which show moderate inter-observer agreement (κ = 0.4–0.6) between graders or modalities in detecting fibrosis (85, 86). Our findings reinforce previous reports (85); we observed that differing operational definitions of fibrosis across studies likely inflate between-study variability and may weaken the observed correlations between scar tissue and vision. This highlights the importance of establishing a consensus OCT-guided definition and a graded severity scale for fibrosis, thereby improving the reliability of future research and clinical trials.

There is a growing need for OCT-anchored, multimodal, compartment-aware definitions to harmonize fibrosis reporting and enable cross-study comparability. A consensus classification system should account for the compartment and extent of fibrotic tissue, distinguishing, for instance, fibrosis above the RPE (subretinal) from fibrosis beneath the RPE (sub-RPE), or mixed lesions involving both, as these configurations may carry different prognoses and therapeutic implications. Indeed, the evidence presented in this review supports previous assertions that fibrotic tissue involving the subretinal space is more frequently associated with photoreceptor disruption and worse visual outcomes than fibrous tissue confined beneath the RPE (7, 80). Although these observations are likely influenced by phenotype-associated confounding, including lesion subtype, foveal involvement, and coexisting atrophy.

Accordingly, future studies and grading systems could adopt consistent descriptors, perhaps denoting sub-RPE scars as “Type 1 fibrosis” and subretinal scars as “Type 2 fibrosis” by analogy to MNV lesion types, to ensure uniform reporting (85). Accordingly, grading should record both the retinal compartment and the location relative to the fovea (subfoveal vs juxtafoveal/extrafoveal, plus central-subfield area), as foveal scarring and central outer-retinal disruption show large, independent associations with worse visual acuity. Consensus criteria should also distinguish between early fibrotic changes and mature scarring, as potential interventions may aim to halt fibrosis in its early, partially reversible phase.

In addition to clarifying definitions, developing quantitative metrics of fibrosis extent is another vital research goal. At present, there is no standardized severity scale for fibrosis, a gap noted by our review and others. The development of sophisticated imaging biomarkers, particularly SHRM volume quantification and morphological characterization, holds promise for creating predictive risk calculators that could stratify patients according to fibrosis propensity. Future studies should establish reproducible methods to measure the extent of fibrotic scarring, such as standardized protocols to determine the fibrotic lesion area on *en face* fundus images or the fibrotic lesion volume on OCT scans. Emerging artificial intelligence tools could aid these efforts; for example, early deep-learning algorithms have been prototyped to automatically detect and segment subretinal fibrosis on OCT or fundus images, although they will need validation in larger, multicenter datasets (87). With agreed-upon quantification standards, it would be possible to compare the “fibrosis burden” across studies and correlate it more precisely with outcomes. Furthermore, we recommend that interventional trials begin to include fibrosis endpoints in a graded rather than a binary fashion, enabling more nuanced assessment of how partial reductions or increases in fibrosis affect vision.

Overall, the quality of evidence across studies was variable. Secondary analyses of large randomized trials offer strong internal validity; however, as *post hoc* assessments conducted in narrowly selected trial populations, their estimates are not fully generalizable to routine care. Conversely, many real-world cohorts better reflect the breadth of clinical presentations, yet are hampered by confounding, attrition, and non-standardized grading (19, 30, 63, 72, 77). Despite these caveats, our synthesis yields a consistent conclusion: subretinal fibrosis is a frequent sequela of treated nAMD and a major driver of long-term visual decline. In summary, there is a clear research mandate to standardize, quantify, and model fibrosis in nAMD. By doing so, we can accelerate the development of therapies targeting fibrosis and ensure that future studies can meaningfully evaluate their efficacy in preserving vision.

### 4.2 Implications for practice

Given the irreversible nature of fibrotic scarring and its devastating impact on central vision, the clinical emphasis must be on prevention rather than treatment of established scars. This paradigm highlights the importance of maintaining continuous angiogenic suppression through proactive treatment regimens, while simultaneously advancing research into anti-fibrotic agents that target the underlying wound-healing pathways driving scar formation. Accordingly, recent reviews have stressed the need for adjunct anti-fibrotic strategies: Tenbrock et al. (79) outlined key profibrotic pathways (e.g., TGF-β, PDGF, Wnt) as potential targets, and Armendariz and Chakravarthy (81) noted that while anti-angiogenic therapy has significantly reduced the incidence of massive disciform scars, more minor subretinal fibrotic scars still commonly develop and drive long-term vision loss (79, 81). Until such therapies become available, recognition of fibrosis as an end-stage manifestation with a poor visual prognosis should galvanize both practitioners and patients toward strict adherence to treatment and regular monitoring to preserve remaining visual function.

## 5 Conclusion

Accordingly, the primary contribution of this review is not definitive effect estimation, but rather to underscore the urgent need for standardized, OCT-anchored definitions of fibrosis and reproducible quantitative imaging measures in neovascular age-related macular degeneration. Macular fibrosis appears to be a frequent and clinically consequential endpoint of nAMD, accounting for sustained deficits in visual function despite adequate control of exudation. Across study designs, fibrosis emerges early, accumulates over time, and is consistently associated with a loss of 25–30 ETDRS letters at mid- to long-term follow-up. These conclusions are based predominantly on low-certainty evidence and should therefore be interpreted cautiously. Progress has been constrained by heterogeneity in imaging-based definitions and largely qualitative grading, which obscures incidence estimates and blunts structure–function inference. Priority areas are clear: (i) consensus, compartment-aware OCT definitions with graded, quantitative metrics; (ii) prospective, time-updated fibrosis endpoints with appropriate handling of competing risks; and (iii) rigorous structure–function mapping beyond BCVA. Clinically, proactive regimens that minimize disease fluctuations appear preferable, yet fibrosis persists in a substantial minority, underscoring an unmet therapeutic need. Standardized measurement, coupled with trials of anti-fibrotic strategies and careful evaluation of treat-and-extend extension patterns, is now required to translate structural insight into durable preservation of vision.

## Data Availability

The original contributions presented in the study are included in the article/**Supplementary Material**. Further inquiries can be directed to the corresponding authors.
