# Supplementary material for: Imaging and functional correlates of fibrosis in neovascular age-related macular degeneration: a systematic review
Source: Front Ophthalmol (Lausanne). 2026 Feb 24;6:1786309. doi: 10.3389/fopht.2026.1786309 (PMC12971459; doi:10.3389/fopht.2026.1786309)
Supplement: Supplementary file 1 [file DataSheet1.docx]

**SUPPLEMENTAL APPENDIX** ***Imaging and Functional Correlates of Fibrosis in Neovascular Age-Related Macular Degeneration: A Systematic Review***

Kimberly Spooner,^1,2^ Samantha Fraser-Bell,^1,3,4^ Dun Jack Fu,^5,6^ Livia Faes,^5,6^  Francesco Romano,^7^ Mariano Cozzi,^7,8^ Andrew A Chang,^1,9^ Sobha Sivaprasad^5,6^

Table of Contents

[1. Search and Screening Documentation 1](#_Toc213693192)

[**Supplemental Appendix 1.** Details of the Search Strategy 2](#_Toc213693193)

[**Supplemental Appendix 2.** Screening and inclusion/exclusion criteria are detailed, along with examples of excluded studies. 3](#_Toc213693194)

[Supplemental Appendix 3. PRISMA 2020 Main Checklist 7](#_Toc213693195)

[Supplemental Appendix 4. PRISMA Abstract Checklist 10](#_Toc213693196)

[2. Study Characteristics and Methodology 12](#_Toc213693197)

[**Supplemental Table S1.** Detailed list of excluded studies with reasons for exclusion at full-text stage. 12](#_Toc213693198)

[**Supplemental Table S2.** Definitions and Functional Outcomes Associated with Subretinal Fibrosis 17](#_Toc213693199)

[**Supplemental Table S3.** Imaging Modalities Used to Define Subretinal Fibrosis in Included Studies 29](#_Toc213693200)

[**Supplemental Table S4.** Functional Outcomes Associated with Subretinal Fibrosis 30](#_Toc213693201)

[3. Analysis 31](#_Toc213693202)

[**Supplemental Table S5.** Subgroup Analysis 31](#_Toc213693203)

[**Pooled incidence of subretinal fibrosis by lesion subtype and treatment regimen** 32](#_Toc213693204)

[**Supplemental Figure S1.** Forest Plot of Subretinal Fibrosis Incidence by Lesion Subtype and Anti-VEGF regimen 33](#_Toc213693205)

[**Supplemental Figure S2.** Funnel Plot of Studies reporting Fibrosis Incidence 34](#_Toc213693206)

[**Supplemental Figure S3.** Forest Plot of Mean Difference in BCVA between Eyes with Subretinal Fibrosis and Those without. 35](#_Toc213693207)

[**Supplemental Table S6.** Detailed Functional Outcomes by Study 36](#_Toc213693208)

[**Supplemental Table S7.** Inter-rater Reliability Studies for Fibrosis Grading 38](#_Toc213693209)

[4. Risk of Bias and Quality Appraisal 39](#_Toc213693210)

[**Supplemental Table S8.** Risk of bias and quality assessment 40](#_Toc213693211)

[5. Certainty of Evidence (GRADE) 45](#_Toc213693212)

[**Supplemental Table S9.** GRADE Evidence Profile 45](#_Toc213693213)

[**Supplementary Table S10**– Summary of Findings (SoF) – Subretinal Fibrosis in nAMD 47](#_Toc213693214)

##

## **1. Search and Screening Documentation**

### **Supplemental Appendix 1.** Details of the Search Strategy

Databases

PubMed/MEDLINE, Embase (Ovid), Cochrane Library (Trials). No study design limits were applied in the primary strategy. Restricted to English language and date limit 2015 🡪 Current

PubMed (MEDLINE)

( "Fibrosis"[Mesh] OR fibros*[tiab] OR cicatrix[tiab] OR cicatricial[tiab] OR scar*[tiab])

AND ( "Macular Degeneration"[Mesh] OR "Macula Lutea"[Mesh] OR "Choroidal Neovascularization"[Mesh] OR

(age-related[tiab] OR age related[tiab] OR senile[tiab]) AND (macula*[tiab] OR macular[tiab] OR retina*[tiab]) AND (degeneration[tiab] OR disease[tiab]) OR

AMD[tiab] OR ARMD[tiab] OR "age-related macular degeneration"[tiab] OR "wet AMD"[tiab] OR nAMD[tiab] OR

"choroidal neovascular*"[tiab] OR "macular neovascular*"[tiab] OR MNV[tiab])

AND ("Tomography, Optical Coherence"[Mesh] OR "Angiography, Optical Coherence Tomography"[Mesh] OR

"Fluorescein Angiography"[Mesh] OR "Angiography"[Mesh] OR "Retinal Imaging"[Mesh] OR "Photogrammetry"[Mesh] OR

OCT[tiab] OR "optical coherence tomograph*"[tiab] OR SD-OCT[tiab] OR "spectral domain OCT"[tiab] OR TD-OCT[tiab] OR PS-OCT[tiab] OR "polarization-sensitive OCT"[tiab] OR

OCTA[tiab] OR "OCT angiograph*"[tiab] OR SS-OCTA[tiab] OR

FA[tiab] OR "fluorescein angiograph*"[tiab] OR ICGA[tiab] OR ICG[tiab] OR

CFP[tiab] OR "color* fundus"[tiab] OR "colour fundus"[tiab] OR "fundus photograph*"[tiab] OR

FAF[tiab] OR "fundus autofluorescence"[tiab] OR "infrared reflectance"[tiab] OR NIR[tiab]) AND (

subretin*[tiab] OR sub-fove*[tiab] OR subfove*[tiab] OR macular[tiab])

Embase (Ovid)

1 exp fibrosis/ OR (fibros* OR cicatrix OR cicatricial OR scar*).ti,ab.

2 exp age related macular degeneration/ OR exp macula lutea disease/ OR exp choroidal neovascularization/

3 ((age-related OR age related OR senile) adj3 (macula* OR retinal OR retina* OR macular) adj3 (degeneration OR disease)).ti,ab.

4 (AMD OR ARMD OR "wet AMD" OR nAMD).ti,ab.

5 ("choroidal neovascular*" OR "macular neovascular*" OR MNV).ti,ab.

6 2 OR 3 OR 4 OR 5

7 exp optical coherence tomography/ OR exp optical coherence tomography angiography/ OR exp fluorescein angiography/ OR exp retinal imaging/ OR exp fundus photography/

8 (OCT OR "optical coherence tomograph*" OR SD-OCT OR TD-OCT OR PS-OCT OR "polarization-sensitive OCT" OR OCTA OR "OCT angiograph*" OR SS-OCTA OR FA OR "fluorescein angiograph*" OR ICGA OR ICG OR CFP OR "colour fundus" OR "color* fundus" OR "fundus photograph*" OR FAF OR "fundus autofluorescence" OR "infrared reflectance" OR NIR).ti,ab.

9 7 OR 8

10 (subretin* OR subfove* OR sub-fove* OR macular).ti,ab.

11 1 AND 6 AND 9 AND 10

12 limit 11 to yr="2005 - 2025"

13 limit 12 to humans

14 remove duplicates from 13

###

### **Supplemental Appendix 2.** Screening and inclusion/exclusion criteria are detailed, along with examples of excluded studies.

***Inclusion Criteria***

Studies were included if they met **all** of the following criteria:

- **Population**
  - Adults with **neovascular age-related macular degeneration (nAMD)** treated with intravitreal anti-VEGF therapy (any agent: ranibizumab, aflibercept, bevacizumab, brolucizumab, conbercept, faricimab).
  - Eyes with treatment-naïve or previously treated nAMD in which **fibrosis**was assessed using imaging modalities (OCT, fundus photography, FAF, OCTA, PS-OCT, or multimodal imaging).
  - Studies including mixed AMD phenotypes were retained if **fibrotic nAMD**eyes formed a distinct subgroup or represented ≥ 40% of the cohort.
- **Intervention / Exposure**
  - Presence, extent, or evolution of **imaging-defined fibrosis**(subretinal or sub-RPE) during or after anti-VEGF therapy.
  - Quantitative or categorical fibrosis measures derived from structural imaging (e.g., SHRM height, HRM area, birefringence volume, or multimodal grading).
  - Studies examining fibrosis as an endpoint, prognostic marker, or component of structural outcomes.
- **Comparators**
  o Eyes without fibrosis (non-fibrotic lesions).
  o Different fibrosis subtypes or locations (subretinal vs sub-RPE).
  o Alternative imaging definitions or quantification thresholds.
  o Different treatment regimens (monthly vs PRN vs treat-and-extend) where fibrosis outcomes were compared.
- **Outcomes**
  At least one of the following outcomes was reported:
  - Best-corrected visual acuity (BCVA, ETDRS letters or logMAR).
  - Retinal sensitivity (microperimetry).
  - Contrast sensitivity, reading speed, or patient-reported visual function.
  - Associations between fibrosis and visual or functional decline (e.g., OR, HR, regression models).
  - Secondary anatomical correlates (EZ/ELM integrity, PED morphology) linked to function.
- **Study Design**
  Eligible designs included:
  - Randomised controlled trials with fibrosis or scarring endpoints (e.g., CATT, IVAN, TREND, AVENUE).
  - Prospective or retrospective cohort studies.
  - Large registry or multicentre real-world analyses (e.g., FRB!, ANZ, Swiss).
  - Interventional case series with ≥ 10 eyes and quantitative imaging assessment

***Exclusion Criteria***

Studies were excluded if they met any of the following criteria:

- Case reports or series with < 10 participants or eyes.
- Review articles, editorials, or commentaries without original imaging data.
- Conference abstracts without accessible full text or quantifiable fibrosis outcomes.
- Animal, histopathological, or in-vitro studies.
- Non-neovascular AMD (dry/atrophic) or other macular diseases (DME, RVO, PCV) unless nAMD fibrosis subgroup was extractable.
- Duplicate or overlapping cohorts—only the most comprehensive or recent dataset retained.

***Screening Process***

Screening was performed in **Covidence** (Veritas Health Innovation, Melbourne, Australia) using a two-stage, independent process.

1. Title and Abstract Screening

Two reviewers independently screened all database records (n = 328 before deduplication). Automated and manual duplicate detection removed 96 records (65 via Covidence, zero manual). Articles clearly unrelated to fibrosis in nAMD or without imaging outcomes were excluded.

2. Full-Text Screening

Full texts of 105 potentially eligible studies were retrieved and reviewed independently by two reviewers.

Disagreements were resolved by consensus or through a third-party review.

Mixed-phenotype AMD studies were included if fibrosis data were separately extractable.

***Exclusion at Full-Text Review***

| **Reason for Exclusion** | **Number of Studies** |
| --- | --- |
| Pre-2015 publication | 15 |
| Review / commentary | 7 |
| Wrong or absent outcomes (no fibrosis–function link) | 6 |
| Wrong indication / non-nAMD population | 3 |
| Wrong study design or interventional focus | 2 |
| Non-English language | 11 |
| Case reports (<10 eyes) | 2 |
| **Total excluded at full text** | **47** |

***Final Inclusion***

Final numbers are reflected in **Figure 1 (PRISMA Flow Diagram).**

***Summary of Screening Flow***

| **Stage** | **Records (n)** |
| --- | --- |
| Identified through databases | **329** |
| — Scopus | 236 |
| — PubMed | 68 |
| — Embase | 25 |
| — Cochrane | 31 |
| Duplicates removed | 96 |
| Total screened (title/abstract) | 233 |
| Excluded at title/abstract | 127 |
| Full texts assessed | 106 |
| Excluded at full text | 47 |
| **Included in qualitative synthesis** | **58** |
| Ongoing / awaiting classification | 0 |

###

### Supplemental Appendix 3. PRISMA 2020 Main Checklist

The following checklist outlines compliance with PRISMA 2020 criteria for systematic reviews and meta-analyses.

| **Section and Topic** | **Item #** | **Checklist item** | **Location where item is reported** |
| --- | --- | --- | --- |
| **TITLE** | | |  |
| Title | 1 | Identify the report as a systematic review. | Title Page |
| **ABSTRACT** | | |  |
| Abstract | 2 | See the PRISMA 2020 for Abstracts checklist. | Manuscript (MS) |
| **INTRODUCTION** | | |  |
| Rationale | 3 | Describe the rationale for the review in the context of existing knowledge. | MS -Introduction |
| Objectives | 4 | Provide an explicit statement of the objective(s) or question(s) the review addresses. | MS - Introduction |
| **METHODS** | | |  |
| Eligibility criteria | 5 | Specify the inclusion and exclusion criteria for the review and how studies were grouped for the syntheses. | MS – Methods -Eligibility Criteria |
| Information sources | 6 | Specify all databases, registers, websites, organisations, reference lists and other sources searched or consulted to identify studies. Specify the date when each source was last searched or consulted. | MS – Methods – Search Strategy |
| Search strategy | 7 | Present the full search strategies for all databases, registers and websites, including any filters and limits used. | Supplemental Appendix – Search Strategy |
| Selection process | 8 | Specify the methods used to decide whether a study met the inclusion criteria of the review, including how many reviewers screened each record and each report retrieved, whether they worked independently, and if applicable, details of automation tools used in the process. | MS – Methods- Study Selection |
| Data collection process | 9 | Specify the methods used to collect data from reports, including how many reviewers collected data from each report, whether they worked independently, any processes for obtaining or confirming data from study investigators, and if applicable, details of automation tools used in the process. | MS – Methods-Data Extraction |
| Data items | 10a | List and define all outcomes for which data were sought. Specify whether all results that were compatible with each outcome domain in each study were sought (e.g. for all measures, time points, analyses), and if not, the methods used to decide which results to collect. | MS -Methods – Definitions + Outcomes |
|  | 10b | List and define all other variables for which data were sought (e.g. participant and intervention characteristics, funding sources). Describe any assumptions made about any missing or unclear information. | Manuscript – Methods, Data Extraction |
| Study risk of bias assessment | 11 | Specify the methods used to assess risk of bias in the included studies, including details of the tool(s) used, how many reviewers assessed each study and whether they worked independently, and if applicable, details of automation tools used in the process. | Manuscript – Methods, Risk of Bias Assessment |
| Effect measures | 12 | Specify for each outcome the effect measure(s) (e.g. risk ratio, mean difference) used in the synthesis or presentation of results. | Manuscript – Methods, Statistical Analysis |
| Synthesis methods | 13a | Describe the processes used to decide which studies were eligible for each synthesis (e.g. tabulating the study intervention characteristics and comparing against the planned groups for each synthesis (item #5)). | Manuscript – Methods, Statistical Analysis and Table 1 |
|  | 13b | Describe any methods required to prepare the data for presentation or synthesis, such as handling of missing summary statistics, or data conversions. | Manuscript – Methods, Statistical Analysis |
|  | 13c | Describe any methods used to tabulate or visually display results of individual studies and syntheses. | Table 1, Forest Plots |
|  | 13d | Describe any methods used to synthesize results and provide a rationale for the choice(s). If meta-analysis was performed, describe the model(s), method(s) to identify the presence and extent of statistical heterogeneity, and software package(s) used. | Manuscript – Methods, Statistical Analysis |
|  | 13e | Describe any methods used to explore possible causes of heterogeneity among study results (e.g. subgroup analysis, meta-regression). | Manuscript – Methods, Statistical Analysis (subgroup and meta-regression) |
|  | 13f | Describe any sensitivity analyses conducted to assess robustness of the synthesized results. | Manuscript – Methods, Statistical Analysis |
| Reporting bias assessment | 14 | Describe any methods used to assess risk of bias due to missing results in a synthesis (arising from reporting biases). | Not assessed – Not Applicable |
| Certainty assessment | 15 | Describe any methods used to assess certainty (or confidence) in the body of evidence for an outcome. | Manuscript – Methods, Certainty of Evidence |
| **RESULTS** | | |  |
| Study selection | 16a | Describe the results of the search and selection process, from the number of records identified in the search to the number of studies included in the review, ideally using a flow diagram. | Manuscript – Results, Study Selection; Figure 1 (PRISMA Flow Diagram) |
|  | 16b | Cite studies that might appear to meet the inclusion criteria, but which were excluded, and explain why they were excluded. | Supplemental Appendix – Table of Excluded Studies |
| Study characteristics | 17 | Cite each included study and present its characteristics. | Table 1 |
| Risk of bias in studies | 18 | Present assessments of risk of bias for each included study. | Supplemental Appendix – Risk of Bias Tables |
| Results of individual studies | 19 | For all outcomes, present, for each study: (a) summary statistics for each group (where appropriate) and (b) an effect estimates and its precision (e.g. confidence/credible interval), ideally using structured tables or plots. | Forest Plots, Supplemental Figures |
| Results of syntheses | 20a | For each synthesis, briefly summarise the characteristics and risk of bias among contributing studies. | Manuscript – Results (sections for each outcome) |
|  | 20b | Present results of all statistical syntheses conducted. If meta-analysis was done, present for each the summary estimate and its precision (e.g. confidence/credible interval) and measures of statistical heterogeneity. If comparing groups, describe the direction of the effect. | Manuscript – Results; Forest Plots |
|  | 20c | Present results of all investigations of possible causes of heterogeneity among study results. | Manuscript – Results, Meta-regression (FOV vs correlation) |
|  | 20d | Present results of all sensitivity analyses conducted to assess the robustness of the synthesized results. | Manuscript – Results; Supplemental Figures |
| Reporting biases | 21 | Present assessments of risk of bias due to missing results (arising from reporting biases) for each synthesis assessed. | Not assessed – Not Applicable |
| Certainty of evidence | 22 | Present assessments of certainty (or confidence) in the body of evidence for each outcome assessed. | Manuscript – Results, Summary of Findings Tables (Supplemental Appendix) |
| **DISCUSSION** | | |  |
| Discussion | 23a | Provide a general interpretation of the results in the context of other evidence. | Manuscript – Discussion |
|  | 23b | Discuss any limitations of the evidence included in the review. | Manuscript – Discussion |
|  | 23c | Discuss any limitations of the review processes used. | Manuscript – Discussion |
|  | 23d | Discuss implications of the results for practice, policy, and future research. | Manuscript – Discussion |
| **OTHER INFORMATION** | | |  |
| Registration and protocol | 24a | Provide registration information for the review, including register name and registration number, or state that the review was not registered. | Manuscript – Methods, PROSPERO Registration |
|  | 24b | Indicate where the review protocol can be accessed, or state that a protocol was not prepared. | PROSPERO |
|  | 24c | Describe and explain any amendments to information provided at registration or in the protocol. | Not Applicable |
| Support | 25 | Describe sources of financial or non-financial support for the review, and the role of the funders or sponsors in the review. | Manuscript – Funding Statement |
| Competing interests | 26 | Declare any competing interests of review authors. | Manuscript – Competing Interests Statement |
| Availability of data, code and other materials | 27 | Report which of the following are publicly available and where they can be found: template data collection forms; data extracted from included studies; data used for all analyses; analytic code; any other materials used in the review. | Manuscript – Data Availability Statement |

^Abbreviations: MS, manuscript; NA, not available; pg., page; sup App., supplemental appendix.^

### Supplemental Appendix 4. PRISMA Abstract Checklist

| **Topic** | **No.** | **Item** | **Reported?** |
| --- | --- | --- | --- |
| **TITLE** |  |  |  |
| **Title** | 1 | Identify the report as a systematic review. | Yes |
| **BACKGROUND** |  |  |  |
| **Objectives** | 2 | Provide an explicit statement of the main objective(s) or question(s) the review addresses. | Yes |
| **METHODS** |  |  |  |
| **Eligibility criteria** | 3 | Specify the inclusion and exclusion criteria for the review. | Yes |
| **Information sources** | 4 | Specify the information sources (e.g., databases, registers) used to identify studies and the date when each was last searched. | Yes |
| **Risk of bias** | 5 | Specify the methods used to assess risk of bias in the included studies. | No |
| **Synthesis of results** | 6 | Specify the methods used to present and synthesize results. | Yes |
| **RESULTS** |  |  |  |
| **Included studies** | 7 | Give the total number of included studies and participants and summarize relevant characteristics of studies. | Yes |
| **Synthesis of results** | 8 | Present results for main outcomes, preferably indicating the number of included studies and participants for each. If meta-analysis was done, report the summary estimate and confidence/credible interval. If comparing groups, indicate the direction of the effect (i.e., which group is favored). | Yes |
| **DISCUSSION** |  |  |  |
| **Limitations of evidence** | 9 | Provide a brief summary of the limitations of the evidence included in the review (e.g., study risk of bias, inconsistency, and imprecision). | No |
| **Interpretation** | 10 | Provide a general interpretation of the results and important implications. | Yes |
| **OTHER** |  |  |  |
| **Funding** | 11 | Specify the primary source of funding for the review. | NA |
| **Registration** | 12 | Provide the register name and registration number. | Yes |

## **2. Study Characteristics and Methodology**

### **Supplemental Table S1.** Detailed list of excluded studies with reasons for exclusion at full-text stage.

| **Article** | **Include?** | **Reason** |
| --- | --- | --- |
| **Angermann 2022** | ✅ Include | Investigates **automated OCT-based quantification of macular fibrosis** in nAMD; directly relevant to review question. |
| **Arrigo 2020** | ❌ Exclude | Prospective real-world study, not randomized; evaluates anti-VEGF switching strategies, not induction protocols . |
| **Arrigo 2024** | ❌ Exclude | Observational retrospective cohort, not RCT; not primarily comparing induction regimens . |
| **Asani 2024** | ❌ Exclude | Study of **genetic and systemic biomarkers** in AMD; fibrosis not assessed as imaging or functional endpoint. |
| **Balaskas 2019** | ❌ Exclude | Focuses on outcomes of switching therapy and treat-and-extend regimens, not induction; not an RCT . |
| **Barikian 2015** | ✅ Include | Prospective randomized pilot study of induction regimens (biweekly vs monthly vs immediate PRN) in treatment-naïve nAMD. |
| **Bilgic 2021** | ✅ Include | Examines single injection responders in nAMD. While fibrosis is listed as a baseline characteristic, fibrosis outcomes are **not the main focus**, and functional correlation with fibrosis is absent. |
| **Bleidißel 2025** | ❌ Exclude | Focuses on OCT angiography features in nAMD but descriptive/diagnostic only, no outcomes on fibrosis or treatment response. |
| **Brown 2015** | ❌ Exclude | Reviews genetic/biologic pathways in AMD, no patient-level treatment or fibrosis outcomes. |
| **Cao 2024** | ✅ Include | Evaluates real-world anti-VEGF treatment response in nAMD, reports on fibrosis and vision outcomes. |
| **Casalino 2018** | ✅ Include | Retrospective imaging biomarker study predicting fibrosis/atrophy during anti-VEGF therapy; not an interventional trial of induction regimens. |
| **Casalino 2020** | ✅ Include | Studies hyperreflective foci and fibrosis development in nAMD eyes under anti-VEGF. Relevant clinical outcomes. |
| **Chakravarthy 2015 (IVAN trial)** | ✅ Include | Large RCT comparing ranibizumab vs bevacizumab and continuous vs discontinuous regimens, with outcomes including GA/fibrosis and vision. |
| **Chandak 2025** | ✅ Include | Real-world study on switching from aflibercept to faricimab in nAMD; evaluates treatment outcomes and relevant to anti-VEGF/fibrosis progression. |
| **Chandra 2024** | ✅ Include | Large multicentre PRECISE study on treatment-naïve nAMD, analysing OCT biomarkers (IRF, SRF, SHRM, fibrosis, atrophy, CST, EZ/ELM integrity, MNV subtype) in relation to baseline VA. Directly relevant to review on OCT biomarkers in nAMD. |
| **Channa 2015** | ✅ Include | Retrospective case series of nAMD eyes treated with anti-VEGF; evaluates development of macular atrophy and links to subretinal fibrosis. |
| **Charles 2023** | ✅ Include | Prospective evaluation of risk of fibrosis and atrophy in eyes treated for nAMD; directly linked to review protocol scope. |
| **Chen 2022** | ❌ Exclude | review |
| **Cheung 2019** | ✅ Include | Focuses on OCT biomarkers and anti-VEGF outcomes; not specifically fibrosis/atrophy progression. |
| **Csincsik 2025** | ❌ Exclude | Focuses primarily on geographic atrophy (GA) progression and risk factors, not OCT biomarkers in nAMD. Outside scope of review. |
| **Daniel 2018** | ✅ Include |  |
| **Daniel 2019** | ✅ **Include** | Prospective cohort from CATT describing morphological changes (nonfibrotic scars, fibrotic scars, atrophy) and long-term VA outcomes in **nAMD eyes after anti-VEGF therapy.** Directly relevant to fibrosis/scarring outcomes in nAMD. |
| **Datseris 2025** | ❌ Exclude | Broad AI/ophthalmology review; not centred on fibrosis/atrophy endpoints in nAMD. |
| **de la Fuente 2024** | ✅ Include | Original study using machine learning to predict fibrosis and atrophy in nAMD over 36 months. |
| **Dolz-Marco 2017** | ✅ Include | Retrospective case series of 10 eyes with type 2 NV converting into type 1 NV after anti-VEGF, discussing mechanisms to avoid **subretinal fibrosis.** Relevant since it evaluates anti-VEGF effects on lesion morphology and fibrosis risk, though small and observational. |
| **Egger 2022** | ❌ Exclude | Review |
| **Finn 2021** | ✅ Include | Long-term outcomes of anti-VEGF–treated nAMD; evaluates macular atrophy and fibrotic changes |
| **Gelişken 2009** | ❌ Exclude | Focuses on RPE tears after **single bevacizumab injection**; complication study, not long-term fibrosis outcomes. |
| **Gianniou 2015** | ✅ Include | Examines refractory intraretinal/subretinal fluid in nAMD treated with ranibizumab; explicitly assesses fibrosis and atrophy risk in refractory cysts. |
| **Gillies 2020** | ✅ Include | 10-year treatment outcomes from two regions using FRB! registry; reports macular atrophy and subretinal fibrosis as key causes of vision loss. |
| Gonzalez-Buendia 2017 | ✅ Include | Prospective study examining OCT predictors of subretinal fibrosis in nAMD treated with anti-VEGF; directly relevant to fibrosis development. |
| **Gräfe 2020** | ✅ Include | Focuses specifically on **subretinal fibrosis (SRFib)** detection using PS-OCT in AMD and other CNV. Directly relevant to fibrosis/imaging outcomes. |
| **Hoffmann 2020** | ✅ Include | Multicentre study of anti-VEGF therapy in routine practice in Germany; provides real-world effectiveness and safety data. |
| **Ito 2017** | ✅ Include | Examines fibrovascular pigment epithelial detachment regression and subretinal fibrosis after aflibercept in nAMD. |
| **Jackson 2015** | ✅ Include | INTREPID trial; relevant as adjunct to anti-VEGF |
| Jaffe 2019 | ✅ Include | Secondary analysis of a large RCT (HARBOR) evaluating predictors and imaging correlates of fibrosis/atrophy under anti-VEGF. |
| **Janse van Rensburg 2021** | ✅ Include | Shows strong association between Type 2 MNV and outer retinal tubulation with subretinal fibrosis, relevant to fibrosis pathogenesis. |
| **Kim 2018** | ✅ Include | Observational study on subretinal fibrosis in nAMD patients receiving anti-VEGF; relevant clinical outcomes (BCVA, OCT, fibrosis progression). |
| **Kim 2020** | ✅ Include | Investigates subretinal fibrosis development in AMD eyes treated with anti-VEGF, directly relevant to fibrosis/scarring progression. |
| **Kim 2022** | ✅ Include | Investigates multilayered PED morphology in nAMD and its association with fibrotic scar formation and poor visual prognosis. |
| **Laishram 2017** | ❌ Exclude | Observational study of microperimetry in macular diseases (AMD, CME, ERM, PED, CSCR, dystrophy, etc.). |
| **Le 2021** | ✅ Include | Case series showing progression from Type 2 MNV to fibrovascular PED after anti-VEGF, directly relevant to fibrosis development. |
| **Lenhof 2025** | ✅ Include |  |
| **Lindenberg 2025** | ✅ Include |  |
| **Liu 2024** | ✅ Include | Analyses OCT angiography biomarkers in predicting anti-VEGF treatment outcomes in neovascular AMD; fits inclusion criteria. |
| **Llorente-González 2022** | ✅ Include | Prospective OCT-based study of retinal/choroidal biomarkers in neovascular AMD patients treated with anti-VEGF; relevant to prognosis and treatment response. |
| **Maruyama-Inoue 2018** | ✅ Include | Evaluates SHRM components classified by OCTA and their prognostic impact on visual outcomes after anti-VEGF in nAMD. |
| Miere 2015 | ✅ Include | Imaging descriptive OCTA study; no interventional design, fibrosis reported morphologically but not as trial outcome. |
| **Moroz 2017 (#256)** | ❌ Exclude | Covers retinal detachment and proliferative vitreoretinopathy, not AMD or subretinal fibrosis. |
| **Motschi 2021 (#257)** | ✅ Include | Develops a PS-OCT algorithm to detect and quantify subretinal fibrosis in neovascular AMD patients. |
| **Ohayon 2020 (#261)** | ✅ Include | Analyses morphological changes of fibrovascular PEDs with a fibrotic component after anti-VEGF in AMD. |
| **Okeagu 2021** | ✅ Include | AREDS2 report analysing causes of poor visual outcome (atrophy vs fibrosis) after anti-VEGF in nAMD; large prospective dataset, directly relevant. |
| **Papavasileiou 2015** | ✅ Include | Observational study of fibrotic scar formation in treated nAMD, evaluates OCT/FAF imaging features and links to visual outcomes. |
| **Querques 2020** | ✅ **Include** | Focuses on **predictors and functional outcomes** of subretinal fibrosis in nAMD patients under anti-VEGF. Directly relevant. |
| **Ramtohul 2022** | ✅ Include | Cohort study on predictors of subretinal fibrosis after anti-VEGF therapy in nAMD; relevant functional and imaging outcomes assessed. |
| **Roberts 2016** | ✅ **Include** | Imaging methodology paper — identifies and quantifies fibrosis with PS-OCT. Relevant to fibrosis detection/characterization. |
| **Roberts 2019** | ✅ **Include** | Focuses on **multimodal imaging (OCT, FAF, en face OCT, fluorescein/ICG)** to evaluate **fibrosis and atrophy in nAMD;** directly relevant to fibrosis detection and imaging biomarkers. |
| **Roberts 2021** | ✅ **Include** | Compares **with vs. without fibrosis** groups. Gives mechanistic/structural insight into fibrosis. Directly relevant. |
| **Roberts 2022** (#55) | ✅ Include | AMD cohort, OCT features associated with treatment response, fits inclusion criteria. |
| **Romano 2022** | ✅ **Include** | Investigates **OCT-based biomarkers (fibrosis, hyperreflective foci, ORT)** and correlates them with **visual outcomes** in treated nAMD. Strong fit with protocol. |
| **Romano 2023** | ✅ Include | Directly evaluates **OCT biomarkers of subretinal fibrosis** in nAMD, correlation with functional outcomes included. |
| **Ryu 2016** | ❌ Exclude | Focused on **treatment patterns and outcomes** in Korean nAMD cohort; fibrosis not assessed as endpoint. |
| Schranz 2024 | ✅ Include | Imaging and functional correlation of fibrosis in nAMD. |
| Souied 2020 | ✅ Include | OCT study of fibrosis in nAMD, describes progression and risk. |
| **Tan 2024** | ✅ Include | Reports on retinal fibrosis in neovascular AMD; relevant outcomes on disease progression and anti-VEGF treatment. |
| **Tenbrock 2022** | ❌ Exclude | Review article |
| **Teo 2020** | ✅ Include | Large registry study on prevalence, incidence, and risk factors for **subretinal fibrosis in** nAMD treated with anti-VEGF. |
| **Teo 2024** (#305) | ✅ Include | Clinical study on OCT biomarkers in AMD, human patients, relevant outcomes. |
| **Toth 2019** (#63) | ✅ Include | Clinical study within CATT, human OCT analysis in AMD |
| **Weigelt 2021** (#315) | ❌ Exclude | Animal model (mice, AAV-mediated cytokine expression). Preclinical, not human. |
| **Willoughby 2015** (#64) | ✅ Include | CATT dataset, OCT subretinal hyperreflective material in AMD |
| **Wu 2022**(#65) | ✅ Include | Focuses on OCTA imaging and fibrosis development in neovascular AMD. Imaging–fibrosis–visual function relationship |
| **Yu 2023** | ✅ Include | Post-hoc AVENUE trial analysis; OCT study of **hyperreflective material and fibrosis biomarkers**in nAMD. |
| **Zhao 2021**(#68) | ✅ Include | Retrospective study of delayed anti-VEGF treatment during COVID-19; reports increased **sub-macular scarring** and worse BCVA. Fits fibrosis + visual function outcomes. |

### **Supplemental Table S2.** Definitions and Functional Outcomes Associated with Subretinal Fibrosis

Study-specific criteria for fibrosis, imaging modalities used, and associated functional outcomes. Where available, mean BCVA (letters) ± SD are reported separately for fibrosis and non-fibrosis groups. Effect sizes and adjustment variables are extracted from multivariable analyses when provided.

| **Study ID (Author, Year)** | **Imaging Modality (OCT, FAF, PS-OCT, OCTA, multimodal)** | **Fibrosis Definition (criteria used)** | **Fibrosis Location** | **Quantification Method (manual, area, AI-based, etc.)** | **Outcome Measure (BCVA letters, logMAR, CS, MP, PROs)** | **Fibrosis Group: Mean BCVA (letters) ± SD** | **Non-Fibrosis Group: Mean BCVA (letters) ± SD** | **Effect Size (OR, HR, β, etc.)** | **Adjustment Variables (if multivariate)** | **Notes / Comments** |
| --- | --- | --- | --- | --- | --- | --- | --- | --- | --- | --- |
| **Angermann et al., 2022^36^** | OCT, CF | Subfoveal fibrosis end-stage | Subfoveal | Clinical grading | BCVA | Nonpersistent: VA 0.92 ± 0.57 logMAR, 5% fibrosis | Persistent: VA 0.58 ± 0.35 logMAR, 1.2% fibrosis | 4× higher risk fibrosis in nonpersistent | Adjusted for persistence | Nonpersistance main driver of fibrosis risk |
| **Barikian et al., 2015^53^** | OCT, FA | “Subretinal fibrosis” noted clinically on follow-up (no formal scale) | Subretinal | Incidence by treatment arm (manual clinical grading) | BCVA letters; CRT; fluid-free interval | NR (reported mean change +1.8 letters in the 6 scarred eyes at 12 mo) | NR | Fibrosis incidence: 20% in q2w arm vs 0% in others; P=0.003 | Age/sex used when testing fluid-free interval (not fibrosis) | Randomized pilot; biweekly induction showed higher fibrosis without VA/CRT benefit. |
| **Bilgic et al., 2021^54^** | SD-OCT | Collagen buildup in retina/subretinal/sub-RPE space | Subfoveal / subretinal | Manual grading | BCVA, CRT | Better baseline VA (20/45) when no fibrosis | Worse when fibrosis present | OR not reported | Multivariate (membrane size, blood, fibrosis absence) | Early presentation & aflibercept use favoured fibrosis-free resolution |
| **Cao et al., 2024^37^** | OCT, FA, ICGA, OCTA | Subretinal fibrosis by multimodal imaging | Subretinal (macular) | Cytokine assays, OCT SHRM thickness | BCVA logMAR, cytokines | SRFi VA worse (~1.0 logMAR) | Non-SRFi VA better (~0.65 logMAR) | Correlation: VEGF, bFGF, TGF-α ↑ fibrosis | Correlation analysis (IL-6, VEGF, bFGF) | Cytokine levels linked to SHRM thickness & VA |
| **Casalino et al., 2018^35^** | Multimodal: OCT, CF, NIR, FA, ICG | Macular scar graded **fibrotic vs non-fibrotic** per CATT photographic definitions | Macular (location by multimodal imaging) | Categorical severity grades; manual multimodal grading; stats via ordinal regression | BCVA letters at M12; MA presence/severity | NR (directional: fibrotic scar ↓BCVA) | NR | HRM thickness & width → fibrotic scar (P<0.001; P=0.02). Fibrotic scar negatively affected BCVA at M12 (P=0.001). | Ordinal/GLM models including HRM metrics, RPD, etc. | Well-defined HRM associated with later scar; study did **not** quantify scar area. |
| **Chakravarthy et al., 2015 (IVAN)^19^** | OCT, FA, CFP | Fibrotic scar (CATT/IVAN criteria) | Subretinal | Reading centre grading | BCVA, GA | NR by fibrosis | NR | OR 1.47 for GA with continuous dosing | Cox regression: drug, regimen | Continuous dosing ↑ GA; fibrosis rates consistent with CATT |
| **Chandak et al., 2025^38^** | SD-OCT | Fibrosis, SHRM, EZ/ELM loss | Subfoveal | Logistic regression (predictors) | BCVA ≥68 or <54 letters | Poor VA <54 linked with fibrosis | Better VA without fibrosis | OR significant | Adjusted for age, baseline VA, OCT signs | Large real-world dataset |
| **Chandra et al., 2024 (PRECISE)^68^** | OCT (Spectralis) | **Foveal fibrosis** graded on OCT (presence/absence) | Foveal | Manual reading-centre grading; multivariable GEE analysis | Presenting VA (ETDRS letters) | NR | NR | OR for poor VA (<54 letters): foveal fibrosis OR 3.85(P<0.001); also, SHRM, IRF, EZ/ELM loss, ↑CST associated. | Multivariable GEE incl. CST, IRF/SRF, SHRM, MNV subtype (PCV), EZ/ELM integrity, SDD, age/sex/ethnicity. | Cross-sectional at baseline (no follow-up VA); highlights strong association of **foveal** fibrosis with poor presenting VA. |
| **Channa et al., 2015^55^** | SD-OCT, FA | Atrophy with/without subretinal fibrosis | Macula (atrophy zones) | Area measurement (mm²/year) | BCVA, atrophy rate | VA decline in fibrosis/atrophy zones | Stable in preserved areas | Mean atrophy enlargement 0.7 ± 0.8 mm²/yr | Risk factors: RAP, monthly dosing, ranibizumab | Fibrosis + atrophy co-localized with CNV regression |
| **Charles et al., 2023^40^** | OCT, CF | Subretinal fibrosis at 5y | Subretinal | Clinical grading | BCVA ETDRS | 46% developed fibrosis → worse VA | Non-fibrosis → better VA (≥70 letters in 53%) | Not given | N/A | Atrophy 60%, fibrosis 46% at 5y |
| **Cheung et al., 2019^41^** | OCT, FA, ICGA | Fibrosis via SHRM composition | Subretinal | Reading centre grading | VA logMAR | Worse VA if fibrosis present (0.71) | Better VA if absent | P<0.001 | Multivariate: SHRM, VA, PCV | SHRM 82.5% fibrosis composition |
| **Daniel et al., 2018 (CATT)^13^** | CFP, FA, OCT | Fibrotic scar (CATT) | Subretinal | Area (disc areas) | VA, scar growth | Scar eyes: VA ↓13 letters (y1–5) | Non-scar: better VA | HR for scar with classic CNV = 4.49; haemorrhage = 2.28 | Cox regression | Scar expanded, atrophy co-localized |
| **Daniel et al., 2019 (CATT)^72^** | CF, FA, OCT (trial imaging) | Focus on non-fibrotic scar (NFS) at Y1; FS per CATT photographic criteria used for outcomes | Macular | Certified reading-centre grading; longitudinal cohort analysis | VA letters at Y1/2/5; FS/NFS/GA/NGA rates | FS group at Y5**:**mean VA 48 letters; NFS group at Y5**:** 73 letters (20/32) | No-scar at Y1**:**62 letters at Y5 (context) | Incidence: FS in NFS eyes 5% at Y2, 28% at Y5; descriptive (no adjusted ORs for VA). | NR for VA models; repeated-measures/GLM used for morphology over time. | NFS eyes maintained good VA through 5 yr; FS associated with worse VA. |
| **de la Fuente et al., 2024^42^** | OCT, clinical + ML | Fibrosis endpoint | Subfoveal/subretinal | ML classifiers (XGB, RF, SVM) | Fibrosis/atrophy at 36m | NR | NR | AUC 0.72 for fibrosis prediction | ETDRS VA strongest predictor | ML feasibility study |
| **Dolz-Marco et al., 2017^22^** | Multimodal: CF, FAF, NIR, FA, SD-OCT; OCTA (subset) | Narrative: Type 2 MNV typically leads to subretinal fibrosis; here, regression to Type 1 after anti-VEGF may mitigate scarring | Subretinal (when fibrosis occurs) | Manual qualitative multimodal/OCTA assessment (no fibrosis area quant) | logMAR BCVA over time | NR (study shows VA improved to ~0.22 logMAR at 3 mo; 0.14 at 12 mo in n=6) | NR | None (case series; no comparative effect size) | NA | Case series (n=10) describing RPE envelopment of Type 2 → Type 1 pattern with better outcomes; fibrosis not measured. |
| **Evans et al., 2020^34^** | SD-OCT (foveal centre-point thickness [FCPT]); CFP/FA for scar grading (reading-centre criteria | Fibrotic scar per CATT/IVAN definitions; incident fibrosis between baseline and 24 months analysed against retinal-thickness variability | Macular lesion (not sub-typed as subretinal vs sub-RPE in this analysis) | Quartile-based analysis of FCPT variability (SD across visits); logistic regression | BCVA (ETDRS letters), incident fibrosis, incident atrophy | NR | **NR** | Fibrosis risk vs lowest quartile (Q1): Q2 OR 1.40 (1.03–1.91); Q3 OR 1.50 (1.10–2.05); Q4 OR 1.95 (1.42–2.68); BCVA difference Q4 vs Q1 = −6.27 letters (95 % CI −8.45 to −4.09) | Adjusted for baseline BCVA, trial (CATT/IVAN), drug, regimen; sensitivity models also included age, lesion size, CNV type, baseline FCPT, and IRF | Post-hoc pooled RCT dataset (CATT + IVAN, n = 1 731 eyes, 24 months); greater thickness variability predicted fibrosis and worse VA across both monthly and PRN arms; fibrosis absent at baseline |
| **Finn et al., 2021 (CATT)^56^** | OCT (TD/SD) + CP/FA; pixel-mapping | FS per CATT photographic criteria; OCT precursors mapped pixel-wise | Localized (pixel-level) | Custom software registration; pixel-wise odds for FS/MA (statistical mapping) | Anatomical endpoints (MA, FS) vs baseline OCT features (not VA) | NA | NA | Localized predictors of **FS**: MNV, SHRM, PED, IRF, sub-RPE fluid (all P<0.05). >75% of FS pixels arose from baseline MNV. | Pixel-level models (ORs for features → FS/MA); not multivariable at eye-level | Study is methodological; demonstrates FS arises where neovascular components existed; SRF not a localized precursor to FS. |
| **Gianniou et al., 2015^57^** | SD-OCT, FA | Fibrosis end-stage in refractory fluid eyes | Subfoveal (35.5%) | Clinical OCT grading | BCVA | Higher fibrosis risk with refractory cysts (OR 3.3) | SRF refractory had better outcomes | OR 3.3 (fibrosis), OR 3.34 (atrophy) | Logistic regression | Refractory cysts worse than SRF for VA/fibrosis |
| **Gillies et al., 2020^12^** | OCT, CF | Subretinal fibrosis noted | Subfoveal | Clinical registry annotation | VA logMAR | Fibrosis main cause of ≥10-letter loss at 10y | Less fibrosis → VA preserved | Not quantified | Region (ANZ vs Swiss) | ANZ better outcomes (more injections, T&E) |
| **Gonzalez-Buendia et al., 2017^43^** | OCT, FA, FAF, CF | Minimum area of fibrosis or atrophy (non-foveal) | Macula (non-foveal) | Fundus + OCT morphology classification | Snellen→logMAR VA | Not explicitly separated; fibrosis vs preserved vs GA compared | Not explicitly separated | Predictive models (AUC 0.635–0.76) | Multivariate incl. age, baseline BCVA, #injections | PRN regimen may have influenced fibrosis development |
| **Gräfe et al., 2020^52^** | PS-OCT | Fibrosis = birefringence of collagen | Subretinal | PS-OCT phase retardation maps | Diagnostic accuracy | NA (focus diagnostic) | NA | Agreement 21/22 eyes | NA | PS-OCT improved diagnosis in doubtful cases |
| **Hoffman et al., n 2020^17^** | OCT, FAF, SS-OCTA | Morphologic fibrosis noted | Macula (foveal vs nonfoveal) | Qualitative OCT + FAF | BCVA, CS, LLVA, reading speed, PRO (NEI-VFQ25) | Not separated numerically | Not separated numerically | Correlation studies only | Adjusted for lesion size/location | Strongest link between CS & patient-reported outcomes |
| **Ito et al., 2017^70^** | OCT, FA, ICGA | Subfoveal fibrosis | Subfoveal | Clinical/OCT grading | BCVA logMAR | Worse (fibrosis) | Better (non-fibrosis) | p<0.01 | CNV subtype (classic vs occult) | 14/61 developed fibrosis, worse outcomes |
| **Jackson et al., 2015 (INTREPID)^58^** | OCT, FA, CF | Fibrosis absence/presence baseline | Macula | Reading centre grading | BCVA, injections, CST | Absence of fibrosis → +5.3 letters vs sham | Presence → less SRT effect | P=0.0284 (VA), P=0.0002 (injections) | Subgroup (lesion ≤4mm, PED) | SRT most effective in actively leaking, non-fibrotic lesions |
| **Jaffe et al., 2019^30^** | OCT, FA, CF | Fibrosis identified by reading centre | Subfoveal / juxtafoveal | Manual grading of CF/OCT | BCVA (ETDRS) | Fibrosis associated with worse long-term BCVA | Non-fibrosis group had better outcomes | Not directly OR; associations reported | Multivariate analysis including lesion size, atrophy | Progressive thinning and lesion size drove vision decline |
| **Janse van Rensburg et al., 2021^69^** | OCT, FA | Fibrosis underlying ORT | Subretinal beneath ORT | OCT visual | ORT development | ORT+fibrosis: poor VA | ORT–fibrosis: better VA | OR for fibrosis with Type 2 MNV = 22.2 | Multivariate: MNV type, size, SRFM | Strong link fibrosis ↔ ORT |
| **Kim et al., 2018^75^** | OCT, FA, ICGA | Fibrotic scar | Subfoveal/subretinal | Clinical reading | BCVA | 5.1% with scars, worse VA | Non-scar better VA | OR for SHRM = 4.6 | Multivariate (SHRM, haemorrhage) | SHRM main predictor |
| **Kim et al., 2020^45^** | OCT, FA, ICGA | Fovea-involving scar | Subfoveal | Clinical imaging | BCVA logMAR | Scar: 1.67 ± 0.58 | GA: 1.18 ± 0.58; none: 0.69 ± 0.64 | p<0.001 | Not detailed | Scars worst prognosis vs GA |
| **Kim et al., 2022^44^** | OCT, FA, ICGA | Hyperreflective band in m-PED (layer 2) | Sub-RPE | Layer thickness measure | BCVA letters | Layer 2: worse VA, ↑fibrosis | No layer 2: improved VA | p=0.009 (VA), p=0.023 (fibrosis) | Regression for VA & fibrosis | Layer 2 = precursor of fibrosis |
| **Le et al., 2021^71^** | SD-OCT | Type 2 MNV → fibrovascular PED | Sub-RPE/subfoveal | OCT progression | BCVA logMAR | VA improved (to ~20/40) | NA | NR | NA | Unusual progression, favourable VA |
| **Lenhof et al., 2025^25^** | SD-OCT, FA | Subretinal fibrosis by MNV subtype | Subretinal | Cumulative incidence | BCVA (ETDRS) | SF at 3y: BCVA ↓ | No SF: better VA | OR by MNV subtype | Multivariate: SHRM, IRF, baseline VA | Type 2 MNV highest fibrosis risk |
| **Lindenberg et al., 2025^46^** | OCT + colour photos | SHRM thickness + reflectivity, fibrosis vs atrophy | Macula | Manual segmentation of OCT | BCVA not collected | Not available | Not available | No effect size calculated | N/A | Fibrosis vs “atrosis” distinction proposed |
| **Liu et al., 2024^59^** | OCT, OCTA, FA | Dense SHRM + FA staining, RPE/EZ/ELM loss | Subretinal (central macula) | ImageJ manual + AngioTool AI (OCTA vessel metrics) | BCVA (logMAR) | Worse baseline BCVA: ~1.00 logMAR | Better baseline BCVA: ~0.65 logMAR | OR for fibrosis: baseline BCVA (0.02), HF (0.11), type 2 MNV (0.08) | Multivariable logistic regression | Quantitative OCTA vessel analysis predictive of fibrosis |
| **Llorente-González et al., 2022^60^** | OCT, FA | Fibrosis (OCT/clinical) | Subretinal/subfoveal | Registry grading | BCVA (ETDRS) | Fibrosis → worse VA | No fibrosis → better VA | OR=8.54 (3y) | Fluid location (IRF ↑, SRF ↓ risk) | IRF predictive of fibrosis, SRF protective |
| **Maruyama-Inoue et al., 2020^47^** | OCT, FA | SHRM with scarring | Subfoveal | Manual grading | BCVA | Worse vision in fibrosis | Better in non-fibrosis | Not reported | None | Limited details, small Japanese cohort |
| **Miere et al., 2015^23^** | FA, OCT, OCTA | FA staining, OCT compact hyperreflective scar >100 µm | Subretinal, under RPE | Qualitative (vascular patterns, thickness) | BCVA (logMAR) | Mean 1.12 ± 0.59 logMAR (≈20/400) | No non-fibrosis control group | OR for vascular patterns (not BCVA) | N/A | Grouped by active vs inactive CNV, but both had fibrosis |
| **Motschi et al., 2021^61^** | PS-OCT | Birefringence-based segmentation | Subretinal | Automated algorithm, 3D lesion area | Lesion area (mm²), agreement with CFP | Not reported | N/A | N/A | N/A | First automated fibrosis quantification method |
| **Ohayon et al., 2020^62^** | OCT, FA, ICGA | Multilayered PED, layer 2 = fibrotic band | Under RPE (PED) | Manual OCT layer thickness measurement | BCVA, OCT thickness | No change in BCVA, but layer 2 = less responsive | N/A | None | N/A | Layer 2 resistant to anti-VEGF, suggests fibrosis |
| **Okeagu et al., 2021 (AREDS2)^48^** | CFP | Subretinal fibrosis (central gradings) | Central macula | Reading centre classification | BCVA (letters) | 14.9 ± 12.3 (~20/500) | 70.1 ± 12.8 (~20/40) | N/A | Multivariate included baseline BCVA, injections | 40% poor vision cases due to fibrosis, 60% atrophy |
| **Papavasileiou et al., 2015^63^** | SD-OCT, FA | Subretinal fibrosis noted (baseline or follow-up) | Subretinal (macula) | Qualitative OCT + fundus grading | BCVA (logMAR), CRT | Worsened eyes: fibrosis noted, VA decline to ~0.26 logMAR → 0.14 final | Improved/stable eyes: better VA (majority) | Not provided | ANOVA on VA/CRT | Small sample; fibrosis linked to worse VA trajectory |
| **Querques et al., 2020^18^** | MC, OCT, FAF, OCTA, microperimetry | Fibrocellular (scar, vessel regression) vs fibrovascular (persistent NV perfusion) | Macula | Manual classification on MC images; OCTA perfusion density | BCVA (logMAR), microperimetry | Fibrocellular: 0.7 ± 0.5 logMAR | Fibrovascular: 0.3 ± 0.2 logMAR (P=0.003) | Perfusion density lower fibrocellular (29%) vs fibrovascular (44%) (P<0.0001) | Adjusted for age/sex | RPE atrophy more common in fibrocellular |
| **Ramtohul et al., 2022^67^** | Multimodal: CF, OCT, FAF, FA, OCTA | SF by CATT definition (CF + OCT) | Subretinal | Reading centre grading + Cox regression | BCVA (logMAR), fibrosis incidence | 77% developed fibrosis by 4 yr; VA returned to baseline (20/118 → 20/103 Snellen) | Eyes without fibrosis had better VA over time | aHR for SF: haemorrhagic BALAD = 2.02; SHRM = 1.83 | Cox multivariate incl. BALAD haemorrhage, SHRM, MNV type | Longest follow-up; high fibrosis risk with BALAD |
| **Roberts et al., 2016^15^** | PS-OCT vs SD-OCT, FA, CF | Subretinal fibrosis as birefringent tissue on PS-OCT | Subretinal / sub-RPE | Automated segmentation algorithm (PS-OCT) | BCVA; fibrosis area mapping | NR (small case series) | NR | Descriptive; PS-OCT identified fibrosis better than SD-OCT | N/A | Proof-of-concept: PS-OCT distinguishes scar vs SHRM components |
| **Roberts et al., 2019^32^** | PS-OCT, SD-OCT | SHRM → fibrosis via birefringence | Subretinal | Manual volumetric (PS-OCT) | BCVA, SHRM volume | ↓ BCVA in fibrosis (exact letters NR) | Better BCVA in non-fibrosis | P values for SHRM reduction (0.002, 0.027) | N/A | Early angiofibrotic switch (3 mo) |
| **Roberts et al., 2021^24^** | PS-OCT, OCTA, SD-OCT, CF | SF = whitish lesion on CF, HRF on OCT, birefringence on PS-OCT | Subretinal (macula) | Automated PS-OCT segmentation + AngioTool vessel analysis | BCVA (ETDRS), OCTA vessel size | SF eyes: larger MNV area, thicker vessels; VA not separated numerically | Non-SF: smaller lesions, better microvasc. | Quantitative OCTA: SF ↑vessel area, junctions, length (P<0.05) | NA | 33% fibrosis rate after >1 yr anti-VEGF |
| **Roberts et al., 2022^31^** | PS-OCT, OCTA, SD-OCT, FA, CF | SF: birefringence on PS-OCT, scar on CF, FA staining | Subretinal | Automated PS-OCT + AngioTool vessel metrics | BCVA (ETDRS), OCTA vessel metrics | Baseline VA worse in SF group (P=0.001) | Better baseline VA in non-SF | SF risk ↑ with IRF (p=0.014), SHRM (p=0.017), worse baseline VA | Logistic regression | After 12 mo, 18% developed SF |
| **Romano et al., 2022^73^** | OCT, FAF, NIR-AF, MP | Fibrosis after RPE tear | Subretinal repair tissue | Qualitative multimodal | BCVA, MP | Worse retinal sensitivity with fibrosis | Better retinal sensitivity if no fibrosis | P=0.03 | Tear size, AF recovery | AF recovery protective |
| **Romano et al., 2023^8^** | OCT, FA, OCTA | Fibrosis sub-RPE (46%), subretinal (23%), mixed (30%) | Subfoveal & parafoveal | Reading centre grading | BCVA (ETDRS) | Mixed/subretinal fibrosis: VA decline −16.4 letters at 10y | Better preserved in sub-RPE fibrosis | HRs for haemorrhage 2.02, injection number 1.01 | Multivariable (CST variation, haemorrhage, injections, baseline VA) | 62.7% fibrosis incidence at 10 years |
| **Schranz et al., 2024^21^** | CFP, SD-OCT, PS-OCT, MP | Fibrosis via multimodal (CFP, OCT, PS-OCT) | Subretinal | Mixed linear model, MP correlation | MP sensitivity (dB) | 7.2–12.6 dB in fibrosis areas | 15–23 dB in non-fibrosis | p<0.001 | Multimodal classification | Multimodal ↑ detection accuracy |
| **Souied et al., 2020^9^** | SD-OCT, FA, CFP | >50% compact HRM | Subretinal/sub-RPE | Manual slice grading | VA, lesion pathway | Not directly quantified | Not directly quantified | Risk ratios (type 2 CNV ↑ fibrosis 4.5–6x) | CNV type, baseline lesion | 3 progression pathways |
| **Tan et al., 2024^64^** | Multimodal + microperimetry | End-stage fibrosis on OCT/CFP | Central macula | Manual lesion marking + MP overlay | Retinal sensitivity (dB), fixation stability | Fibrosis 5.5 ± 5.4 dB | Atrophy 6.2 ± 7.0 dB; normal 27.8 ± 4.3 | Not effect size but mean difference | N/A | Fibrosis had lowest retinal sensitivity among lesions |
| **Teo et al., 2020 (FRB!)^49^** | CFP, OCT, FA | Physician-graded fibrosis | Subfoveal / extrafoveal | Physician grading | BCVA (letters) | Lower vision in fibrosis eyes | Higher vision in non-fibrosis eyes | OR for fibrosis: poor baseline VA 5.3, lesion size 1.08, activity 1.58 | Baseline VA, lesion size, lesion type, activity | Prevalence up to 40.7% at 10 years |
| **Teo et al., 2024^50^** | OCT, CFP, FA | Subfoveal fibrosis: HRM + pallor/FA staining | Subfoveal | Manual OCT/FA correlation | BCVA (letters) | Fibrosis: –1.4 ± 17.1 letters | Non-fibrosis: +6.0 ± 17.4 letters | β: intact EZ/ELM +29.4, HRM width –3.8 | Regression adjusted for lesion type, haemorrhage | Preserved EZ/ELM predicts better VA despite fibrosis |
| **Toth et al., 2019^33^** | SD-OCT, CFP, FA | FS defined by CATT | Subretinal | Pixel-level overlays | OCT features, VA | Worse VA, ↑ lesion thickness (151µm) | Better VA, thinner lesion | P<0.001 | None | Photoreceptor thinning extends beyond FS |
| **Willoughby et al., 2015^16^** | OCT, CFP, FA | SHRM persistence linked to fibrosis | Subretinal (foveal/para) | Manual height/width | BCVA letters | 64% scar if SHRM persistent | 31% scar if SHRM resolved | P<0.0001 | Location (foveal vs non) | SHRM height/width correlated with VA |
| **Wu et al., 2022^65^** | OCTA, OCT | Subretinal fibrosis in NVAMD | Subretinal | OCTA density, manual | BCVA, CMT | No sig BCVA change (NS) | N/A | p=0.03 (fibrosis ↑ 1.21x) | CMT, CNV density | Fibrosis ↑ despite therapy |
| **Yu et al., 2023 (AVENUE)^51^** | OCT | HRM boundary remodelling phenotype | Subretinal HRM zone | Manual grading of OCT boundaries | BCVA (letters), MA risk | Partial/absent HRM-BR: –6.1 letters | Complete HRM-BR: similar to resolved HRM | OR for poor BR: age 0.96, baseline HTC 0.06 | Multivariate: age, baseline HTC | Complete remodelling linked to preserved VA |
| **Zhao et al., 2021^66^** | OCT, OCTA, FA, ICGA | Sub-macular scar | Subretinal | Clinical diagnosis (scar presence) | BCVA | Worse BCVA in delayed group | Stable BCVA in non-delayed | p<0.05 | Delay status, baseline BCVA | COVID-19 delays ↑ fibrosis risk |
| AF = Autofluorescence; AMD = Age-related macular degeneration; ANZ = Australia and New Zealand; aHR = Adjusted hazard ratio; AI = Artificial intelligence; AUC = Area under the ROC curve; BALAD = Bacillary layer detachment; BCVA = Best-corrected visual acuity; BR = Boundary remodelling; CATT = Comparison of Age-related Macular Degeneration Treatments Trials; CF = Colour fundus; CFP = Colour fundus photography; CH = Switzerland; CNV = Choroidal neovascularization; CST = Central subfield thickness; CMT = Central macular thickness; CRT = Central retinal thickness; CS = Contrast sensitivity; dB = Decibels (microperimetry); ELM = External limiting membrane; ETDRS = Early Treatment Diabetic Retinopathy Study; EZ = Ellipsoid zone; FA = Fluorescein angiography; FAF = Fundus autofluorescence; GA = Geographic atrophy; GEE = Generalized estimating equations; GLM = Generalized linear model; HF = Hyperreflective foci (or foci); HR = Hazard ratio; HRF = Hyperreflective foci; HRM = Hyperreflective material; HRM-BR = Hyperreflective material boundary remodelling; HTC = Hypertransmission into choroid; ICGA/ICG = Indocyanine green angiography/Indocyanine green; IR = Infrared reflectance; IRF = Intraretinal fluid; IVAN = Inhibition of VEGF in Age-related choroidal Neovascularisation trial; LLVA = Low-luminance visual acuity; logMAR = Logarithm of the minimum angle of resolution; MA = Macular atrophy; MC imaging = Multicolour imaging; ML = Machine learning; MNV = Macular neovascularization; MP = Microperimetry; NEI-VFQ25 = National Eye Institute Visual Function Questionnaire-25; NFS = Non-fibrotic scar; NIR = Near-infrared reflectance; NIR-AF = Near-infrared autofluorescence; NVAMD = Neovascular age-related macular degeneration; OCT = Optical coherence tomography; OCTA = OCT angiography; OR = Odds ratio; ORT = Outer retinal tubulation; PCV = Polypoidal choroidal vasculopathy; PED = Pigment epithelial detachment; m-PED = Multilayered PED; PRN = Pro re nata (as needed); PROs = Patient-reported outcomes; PS-OCT = Polarization-sensitive OCT; q2w/q4w/q8w = Every 2/4/8 weeks; RCT = Randomized controlled trial; RF = Random forest; RPD = Reticular pseudodrusen; RPE = Retinal pigment epithelium; SD = Standard deviation; SD-OCT = Spectral-domain OCT; SDD = Subretinal drusenoid deposits; SF/SRFi = Subretinal fibrosis / eyes with subretinal fibrosis; SHRM = Subretinal hyperreflective material; SRT = Stereotactic radiotherapy; SS-OCTA = Swept-source OCT angiography; SVM = Support vector machine; TAE = Treat-and-extend; TD-OCT = Time-domain OCT; tAMD = Typical AMD; VA = Visual acuity. | | | | | | | | | | |

###

### **Supplemental Table S3.** Imaging Modalities Used to Define Subretinal Fibrosis in Included Studies

Fibrosis detection criteria, strengths, and limitations are summarised by imaging modality. Representative studies are listed for each modality. Studies using multiple imaging approaches (e.g., OCT + FA/ICGA + OCTA) are counted under each relevant category.

| **Imaging Modality** | **Studies Using** | **Definition Criteria** | **Strengths** | **Limitations** | **Inter-rater Reliability Evidence** | **Reproducibility Evidence (ICCs / Kappa)** |
| --- | --- | --- | --- | --- | --- | --- |
| **OCT** | Willoughby 2015;^16^ Cheung 2019;^41^ Finn 2021;^56^ Chandra 2024;^68^ Lenhof 2025;^25^ Liu 2024;^59^ Teo 2024^50^ | SHRM evolving into dense hyperreflective scar; subfoveal HRM burden; foveal fibrosis (present/absent) | Widely available; layer-resolved; longitudinal tracking | Composition ambiguity; shadowing; scanner heterogeneity | Reading-centre protocols (CATT, PRECISE) with masked graders; adjudication standard | CATT/IVAN: high agreement (kappa not always reported); reproducible fibrosis incidence across centres |
| **FA / ICGA (+/− CFP)** | Daniel 2018;^13^ Jaffe 2019;^30^ Teo 2020;^49^ Romano 2023;^8^ Okeagu 2021;^48^ Zhao 2021^66^ | Early hypofluorescence / late staining; white/yellow plaques on CFP | Historical reference standard; good localization; complements OCT | Limited layer detail; dye risks; subtle lesions variable | Reading-centre frameworks (CATT/IVAN) widely used | Acceptable reproducibility; adjudicated gradings; inter-grader variability higher for subtle scars |
| **PS-OCT** | Roberts 2016;^15^ Roberts 2019;^32^ Roberts 2021;^24^ Roberts 2022;^31^ Motschi 2021;^61^ Gräfe 2020^52^ | Birefringent collagen signal; optic-axis uniformity maps | Tissue-specific; early detection; allows automated quantification | Limited availability; single-centre expertise; not yet standardized | Vienna group: automated segmentation consistent across graders | Roberts/Motschi: ICC >0.85–0.9; Gräfe: agreement 95% (21/22); reproducibility superior to SD-OCT/FA |
| **OCTA** | Miere 2015;^23^ Roberts 2021;^24^ Roberts 2022;^31^ Wu 2022;^65^ Schranz 2024;^21^ Cao 2024^37^ | Flow voids; pruning over scar; vessel metrics (area, length, junctions) | Non-invasive vascular context; quantitative metrics possible | Projection/shadowing artifacts; segmentation errors; metrics not standardized | Semiautomated tools (AngioTool, pixel mapping) improved consistency | Reproducibility modest; no formal ICCs; usually interpreted with OCT/CFP for reliability |
| **Abbreviations** OCT = Optical coherence tomography; FA = Fluorescein angiography; ICGA = Indocyanine green angiography; CFP = Colour fundus photography; PS-OCT = Polarization-sensitive OCT; OCTA = OCT angiography; SHRM = Subretinal hyperreflective material; HRM = Hyperreflective material; ICC = Intraclass correlation coefficient; CATT = Comparison of AMD Treatments Trials; IVAN = Inhibition of VEGF in Age-related choroidal Neovascularisation trial. | | | | | | |

###

### **Supplemental Table S4.** Inter-rater Reliability and Reproducibility of Imaging Based Fibrosis Grading

| **Study (Author, Year)** | **Imaging Modality** | **Grading Criteria** | **Inter-rater Reliability Metric(s)** | **Key Findings** | **Notes** |
| --- | --- | --- | --- | --- | --- |
| **CATT/IVAN Reading Centres (Daniel 2018;^13^ Chakravarthy 2015^19^)** | CFP, FA, OCT | Fibrotic scar per standardized trial definitions (yellow lesion with staining, OCT hyperreflective scar) | Kappa coefficients not always reported; masked grading with adjudication | High reproducibility across centres; consistent fibrosis incidence reporting | Reading centres established “gold standard” approach for fibrosis adjudication |
| **Casalino 2018 (UK tertiary centre)^35^** | Multimodal (OCT, CF, NIR, FA, ICGA) | Macular scar graded as fibrotic vs non-fibrotic | Ordinal regression models; inter-grader agreement not numerically given | Good consistency when HRM width + thickness present; fibrosis grading reproducible | Supports HRM as fibrosis biomarker |
| **Roberts 2016; Roberts 2019; Roberts 2021; Roberts 2022 (Vienna group)^15,24,31,32^** | PS-OCT, SD-OCT, FA, CFP, OCTA | Fibrosis defined by birefringence signal vs conventional criteria | PS-OCT segmentation reproducibility: ICC >0.9; automated birefringence mapping consistent | PS-OCT significantly improved reproducibility vs SD-OCT or FA alone | Early adoption of automated quantification pipelines |
| **Motschi 2021 (Austria)^61^** | PS-OCT | Automated birefringence-based segmentation (lesion area in mm²) | ICC >0.85 (intra/inter-rater) | High reliability for automated fibrosis area measurement | Proof of concept for AI-based reproducibility |
| **Gräfe 2020^52^ (Netherlands)** | PS-OCT vs FA/ICGA | Collagen birefringence vs dye leakage/scarring | Agreement rate 21/22 cases (95%) | PS-OCT more reliable in ambiguous lesions | Small validation cohort |
| **Querques 2020^18^** | Multimodal + OCTA | Fibrocellular vs fibrovascular phenotype | No kappa reported; classification by two graders | Consistent classification across graders, based on perfusion density and imaging phenotype | Phenotypic classification reproducible but less standardized |
| **Schranz 2024^21^** | CFP, OCT, PS-OCT | Fibrosis vs atrophy via multimodal overlays | Mixed linear model; intra-grader consistency strong | Multimodal approach ↑ detection accuracy | Inter-rater reliability not directly quantified |
| **Abbreviations** CFP = Colour fundus photography; FA = Fluorescein angiography; ICGA = Indocyanine green angiography; OCT = Optical coherence tomography; PS-OCT = Polarization-sensitive OCT; SD-OCT = Spectral-domain OCT; OCTA = OCT angiography; HRM = Hyperreflective material; SHRM = Subretinal hyperreflective material; ICC = Intraclass correlation coefficient.  Note: This table summarizes studies assessing inter-rater reliability and reproducibility of imaging-based fibrosis grading. These findings relate to grading consistency and do not establish prognostic validity, biological equivalence of fibrosis definitions, or effect size estimates. | | | | | |

## **3. Analysis**

### **Supplemental Table S5.** Subgroup Analysis

Fibrosis incidence stratified by anti-VEGF regimen and MNV subtype.

| **Subgroup** | **Studies Reporting** | **Findings on Fibrosis Incidence** | **Notes** |
| --- | --- | --- | --- |
| **Fixed monthly dosing** | CATT (Daniel 2018,^13^ Jaffe 2019^30^); IVAN (Chakravarthy 2015^19^) | CATT 2y: fibrosis ~45% (no difference between monthly vs PRN); IVAN: similar fibrosis incidence across regimens | Fixed dosing did not reduce fibrosis, but increased GA risk |
| **PRN (as needed)** | Barikian 2015;^53^ Gonzalez-Buendia 2017;^43^ Gillies 2020 (FRB! registry)^12^ | Barikian: fibrosis 20% in q2w arm vs 0% in others; Gonzalez-Buendia: fibrosis/atrophy >30% at 2y; FRB!: fibrosis ~35–40% over 5–10y | Undertreatment and fewer injections linked to higher fibrosis |
| **Treat-and-extend (T&E)** | Angermann 2022;^36^ Charles 2023;^40^ Ito 2017;^70^ Gillies 2020 (FRB!)^12^ | Angermann: fibrosis 5% in persistent T&E vs 20% in nonpersistent; Charles: 46% fibrosis at 5y; Gillies: T&E cohort had less fibrosis than PRN | T&E protective if persistence maintained |
| **Type 1 MNV** | Souied 2020;^9^ Dolz-Marco 2017^22^ | Souied: fibrosis ~20% at 5y; Type 1 lesions often regress or persist without scarring | Type 1 protective relative to Type 2 |
| **Type 2 MNV** | Romano 2023;^8^ Souied 2020;^9^ Lenhof 2025;^25^ Liu 2024^59^ | Romano: fibrosis 62.7% at 10y; Souied: 45–60% at 5y; Lenhof: highest cumulative fibrosis risk across subtypes | Consistently the strongest risk factor |
| **Type 3 MNV (RAP)** | Channa 2015;^55^ Kim 2020^45^ | Kim: fibrosis ~25–30% at 4y, with higher atrophy rates; RAP often progresses to atrophy with scarring | Co-localization with atrophy common |
| **PCV** | Cheung 2019;^41^ Kim 2018;^75^ Chandra 2024^68^ | Cheung: SHRM fibrosis composition ~82%; Kim: 5.1% fibrosis scars at 1y, higher long-term; PRECISE (Chandra 2024): PCV significant fibrosis predictor | Fibrosis often linked to haemorrhage and SHRM in PCV eyes |
| **Abbreviations** MNV = Macular neovascularization; PCV = Polypoidal choroidal vasculopathy; RAP = Retinal angiomatous proliferation; PRN = Pro re nata (as needed); T&E = Treat-and-extend; SHRM = Subretinal hyperreflective material; GA = Geographic atrophy; VA = Visual acuity. | | | |

### **Pooled incidence of subretinal fibrosis by lesion subtype and treatment regimen**

| **Group Type** | **Group** | **k** | **Pooled %** | **95% CI** | **I²** | **Tau²** |
| --- | --- | --- | --- | --- | --- | --- |
| Subtype | Type 1 | 2 | 15.1 | 14.9–15.2 | 0.0% | 0.000 |
| Subtype | Type 2 | 2 | 61.5 | 22.4–89.8 | 100.0% | 1.522 |
| Subtype | PCV | 2 | 7.5 | 3.7–14.5 | 99.7% | 0.282 |
| Subtype | RAP/Type 3 | 1 | 29.8 | 29.4–30.3 | 0.0% | 0.000 |
| Subtype | Mixed | 4 | 41.0 | 36.4–45.7 | 100.0% | 0.039 |
| Regimen | Monthly | 1 | 40.0 | 39.9–40.1 | 0.0% | 0.000 |
| Regimen | PRN | 3 | 46.6 | 45.0–48.2 | 99.8% | 0.003 |
| Regimen | T&E | 3 | 24.2 | 15.8–35.1 | 100.0% | 0.218 |
| Overall | All subtypes combined | 11 | 29.4 | 25.1–34.1 | 100.0% | 0.132 |

### **Supplemental Figure S1.** Forest Plot of Subretinal Fibrosis Incidence by Lesion Subtype and Anti-VEGF regimen


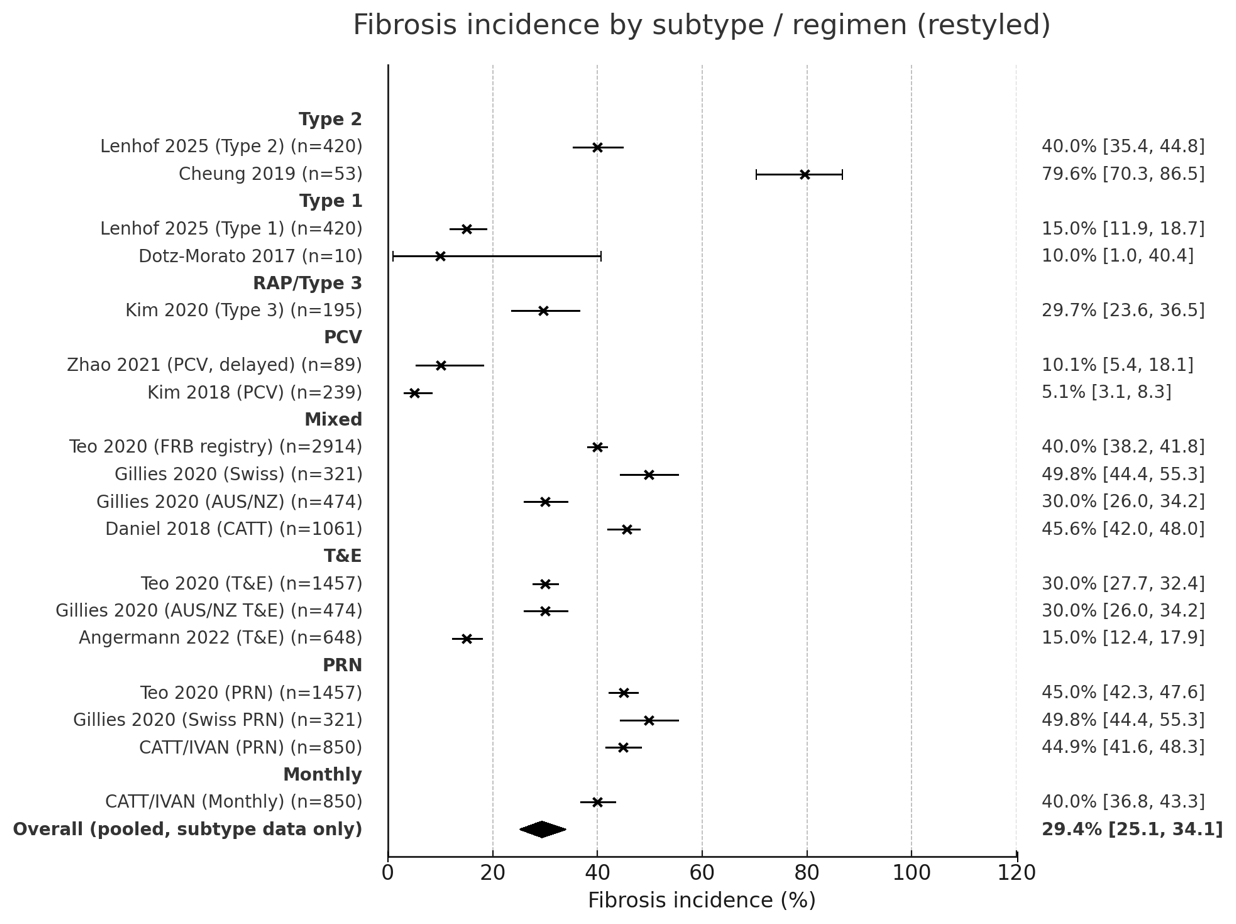


**Figure 2. Forest plot of subretinal fibrosis incidence by lesion subtype and anti-VEGF regimen.**
Each black square represents an individual study’s incidence estimate with 95 % confidence intervals (horizontal black whiskers with capped ends). The size of each square reflects the study weight in the random-effects model. The solid black diamond denotes the pooled random-effects estimate for each subgroup, and the overall pooled estimate across all studies. The x-axis shows fibrosis incidence (%). Between-study heterogeneity was high (I² = 100 %, τ² = 0.132).

### **Supplemental Figure S2.** Funnel Plot of Studies reporting Fibrosis Incidence

**
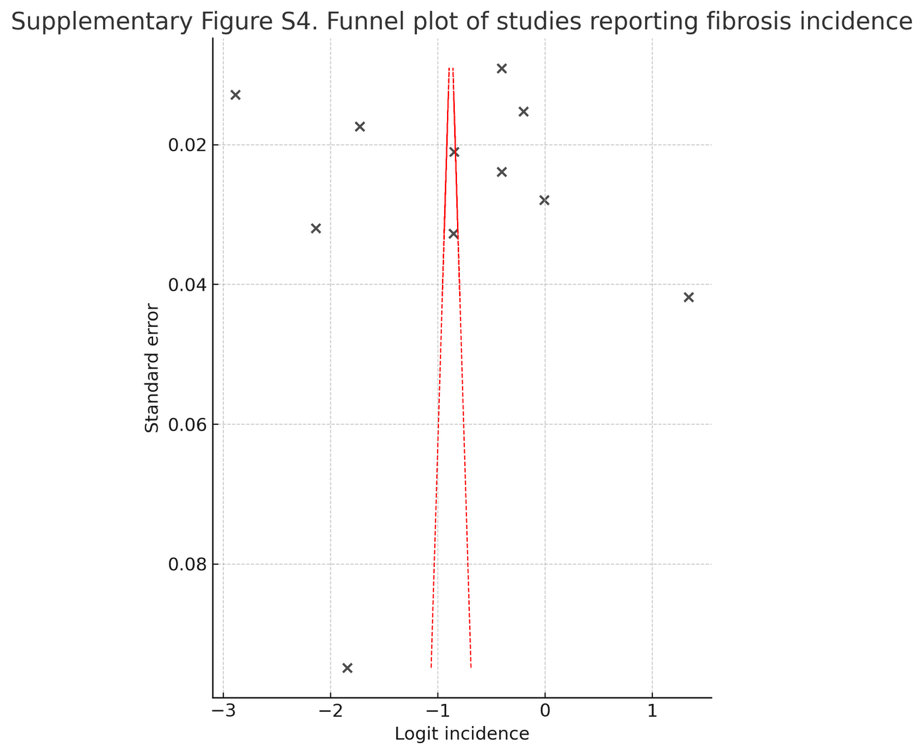
**

**Supplemental Figure S2. Funnel plot of studies reporting subretinal fibrosis incidence.**

**Funnel plot showing the relationship between study precision (standard error) and effect size (logit-transformed incidence). The plot demonstrates roughly symmetrical scatter around the pooled estimate, indicating no evidence of publication bias. Egger’s regression test (p = 0.158) and Begg’s rank correlation test (Kendall’s τ = 0.09, p = 0.76) both suggest no significant small-study effects. Minor asymmetry may reflect between-study heterogeneity rather than selective reporting.**

### **Supplemental Figure S3.** Forest Plot of Mean Difference in BCVA between Eyes with Subretinal Fibrosis and Those without.


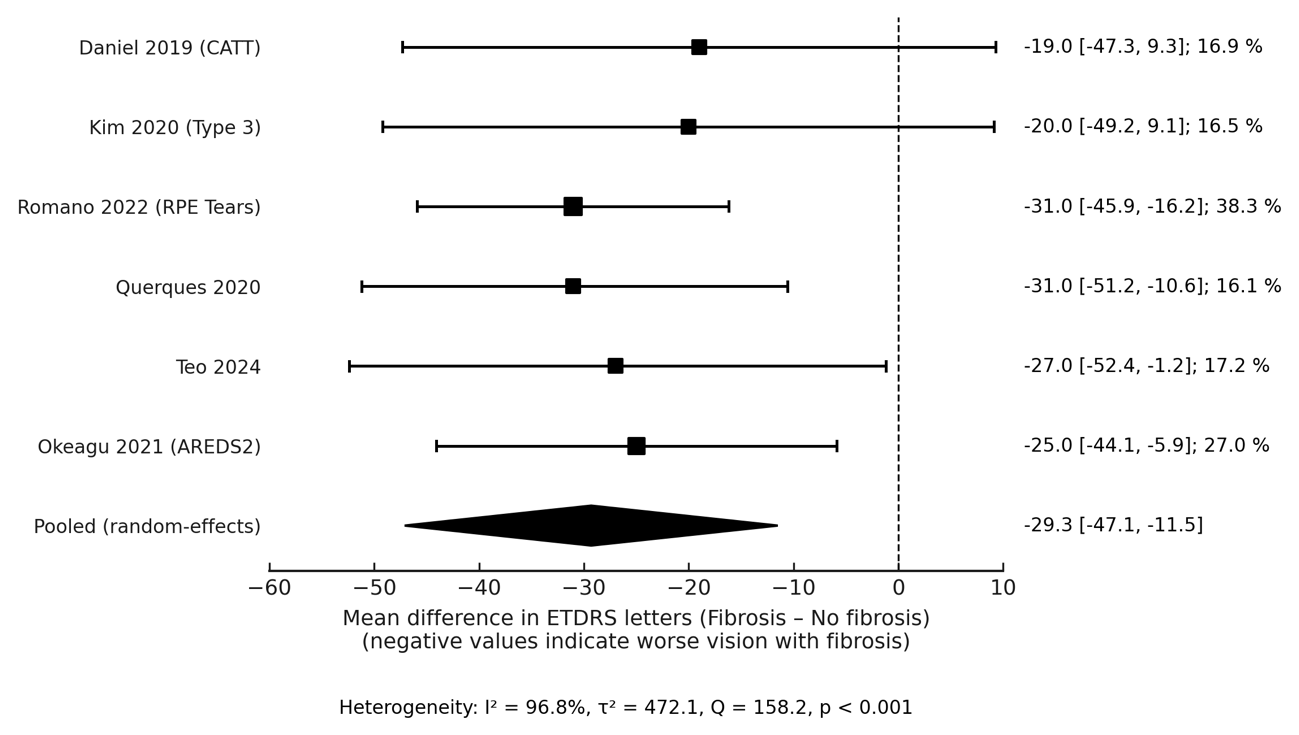


**Forest plot showing the mean difference in best-corrected visual acuity (ETDRS letters) between eyes with subretinal fibrosis and those without.**

**Negative values indicate worse vision in eyes with fibrosis. Individual study estimates with 95% confidence intervals (CIs) are shown as black squares, sized by study weight under a random-effects model (DerSimonian–Laird). The pooled mean difference was −29.3 letters (95% CI −47.1 to −11.5), indicating substantially poorer vision in eyes with fibrosis. Between-study heterogeneity was high (I² = 96.8 %, τ² = 472.1, Q = 158.2, p < 0.001).**

### **Supplemental Table S6.** Detailed Functional Outcomes by Study

Outcomes shown for fibrosis vs. no-fibrosis where available. Negative differences indicate worse function with fibrosis.

| **Study (Author, Year)** | **Country / Setting** | **Sample Size (Fib / No Fib)*** | **Functional Measure** | **Mean ± SD — Fibrosis Group** | **Mean ± SD — Non-Fibrosis Group** | **Absolute Difference** | **p-value** | **Imaging Modality(ies)** | **Follow-up (mo)** | **Notes** |
| --- | --- | --- | --- | --- | --- | --- | --- | --- | --- | --- |
| **Angermann et al., 2022^36^** | Austria | Persistent 614 / Nonpersistent 34 | BCVA (logMAR) | 0.92 ± 0.57 | 0.58 ± 0.35 | −0.34 logMAR (~17 letters) | <0.01 | OCT, CF | 60 | Non-persistence → 4× fibrosis risk |
| **Daniel et al., 2018 (CATT 5y)^13^** | USA, multicentre | Scar group ~500 / non-scar ~560 | BCVA (ETDRS letters) | ↓13 letters (y1–5) | Stable VA | −13 | <0.001 | CFP, FA, OCT | 60 | Fibrosis expansion co-localized with atrophy |
| **Daniel 2019 et al., (CATT NFS analysis)^72^** | USA, multicentre | FS at 5y: 28%; NFS at 1y n=39 | BCVA (ETDRS letters) | FS group: 48 letters | NFS group: 73 letters | −25 | <0.001 | CF, FA, OCT | 60 | NFS eyes maintained good VA |
| **Hoffmann et al., 2020^17^** | Germany | Bilateral nAMD cohort | LLVA, CS, PROs | Worse LLVA & CS; PRO scores lower | Better function | Not quantified | — | OCT, FAF, SS-OCTA, MP | Variable | CS loss most correlated with PROs |
| **Ito et al., 2017^70^** | Japan | 14 fibrosis / 47 non-fibrosis | BCVA (logMAR) | 0.92 ± 0.21 | 0.54 ± 0.17 | −0.38 logMAR (~19 letters) | <0.01 | OCT, FA, ICGA | 24 | Subfoveal fibrosis incidence 23% |
| **Okeagu et al., 2021 (AREDS2)^48^** | USA | 594 with incident nAMD | BCVA (ETDRS letters) | 14.9 ± 12.3 | 70.1 ± 12.8 | −55 letters | <0.001 | CFP (central grading) | 24 | Fibrosis cause in ~40% of vision loss cases |
| **Papavasileiou et al., 2015^63^** | UK | 26 eyes | BCVA (logMAR) | Decline to ~0.26 logMAR | Improved/stable ~0.14 logMAR | −0.12 logMAR (~6 letters) | <0.05 | SD-OCT, FA | 14 | Small sample, retrospective |
| **Querques et al., 2020^18^** | Italy | 41 eyes (fibrocellular vs fibrovascular) | BCVA (logMAR), MP (dB) | 0.7 ± 0.5; 15.2 dB | 0.3 ± 0.2; 21.1 dB | −0.4 logMAR; −6 dB | 0.003 | OCT, FAF, OCTA, MP | Cross-sectional | Fibrocellular phenotype worse |
| **Romano et al., 2022^73^** | Italy | 48 (RPE tears, repair tissue vs non) | MP (dB) | Worse sensitivity with fibrosis | Better sensitivity | ~−5 dB | 0.03 | OCT, FAF, MP | ≥12 | Atrophy recovery protective |
| **Romano et al., 2023^8^** | Italy, Singapore | Mixed fibrosis subtypes; 10y | BCVA (ETDRS letters) | Subretinal/mixed: −16.4 letters | Sub-RPE fibrosis: milder loss | −16.4 (vs preserved) | 0.002 | OCT, FA, CF, OCTA | 120 | Haemorrhage & Type 2 MNV ↑ risk |
| **Schranz et al., 2024^21^** | Austria | 30 eyes | MP (dB) | 7–12 dB | 15–23 dB | −8 to −11 dB | <0.001 | CFP, SD-OCT, PS-OCT, MP | ≥12 | Fibrosis zones had lowest sensitivity |
| **Tan et al., 2024^64^** | Singapore | 140 eyes AMD / 66 controls | MP (dB) | 5.5 ± 5.4 | Atrophy: 6.2 ± 7.0; Normals: 27.8 ± 4.3 | −1 dB vs atrophy; −22 dB vs normals | <0.001 | OCT, OCTA, IR, CFP, MP | Cross-sectional | Fibrosis worst sensitivity |
| **Abbreviations** BCVA = Best-corrected visual acuity; ETDRS = Early Treatment Diabetic Retinopathy Study; VA = Visual acuity; logMAR = Logarithm of the minimum angle of resolution; MP = Microperimetry; dB = Decibels; LLVA = Low luminance visual acuity; CS = Contrast sensitivity; PROs = Patient-reported outcomes; CFP = Colour fundus photography; FA = Fluorescein angiography; ICGA = Indocyanine green angiography; OCT = Optical coherence tomography; OCTA = OCT angiography; FAF = Fundus autofluorescence; IR = Infrared reflectance; PS-OCT = Polarization-sensitive OCT. | | | | | | | | | | |

###

### **Supplemental Table S7.** Functional Outcomes Associated with Subretinal Fibrosis

Summary of functional outcomes reported across included studies. Subretinal fibrosis was consistently associated with worse vision and retinal function across multiple measurement methods. Negative values represent worse function in the fibrosis group compared with eyes without fibrosis.

| **Functional Measure** | **Studies** | **Key Findings** | **Avg. Difference (Fibrosis vs No Fibrosis)** |
| --- | --- | --- | --- |
| **BCVA (ETDRS letters)** | Daniel 2018 (CATT 5y);^13^ Daniel 2019 (CATT);^72^ Romano 2023;^8^ Angermann 2022;^36^ Teo 2024;^64^ Okeagu 2021 (AREDS2)^48^ | Fibrosis consistently linked to reduced VA; CATT: scarred eyes lost ~13 letters over 5y; Romano: mixed/subretinal fibrosis −16.4 letters at 10y; Angermann: nonpersistent therapy ↑ fibrosis with poorer VA; Teo 2024: fibrosis eyes lost ~7.4 letters compared to non-fibrosis | ≈ −10 to −15 letters (long-term), up to −16.4 letters in mixed fibrosis |
| **Microperimetry (dB)** | Querques 2020;^18^ Romano 2022 (RPE tear cohort);^73^ Schranz 2024;^21^ Tan 2024^64^ | Fibrosis associated with lower retinal sensitivity; Querques: fibrocellular fibrosis 0.7 logMAR and lower MP sensitivity; Romano: fibrosis after RPE tear = worse MP sensitivity (P=0.03); Schranz: fibrosis zones 7–12 dB vs 15–23 dB in preserved; Tan: fibrosis 5.5 ± 5.4 dB vs 27.8 ± 4.3 in normals | ≈ −8 to −15 dB reduction |
| **Low Luminance VA (LLVA)** | Hoffmann 2020 (bilateral nAMD, cross-sectional)^17^ | Fibrosis correlated with impaired LLVA alongside worse BCVA and CS | Not quantified separately; qualitative decline only |
| **Contrast Sensitivity (CS)** | Hoffmann 2020;^17^ Schranz 2024 ^21^ (indirect, MP correlation) | Fibrosis linked with poorer CS; strongest correlation between CS and NEI-VFQ25 patient-reported outcomes; Schranz: MP loss parallels CS loss | Approx. −0.3 to −0.5 logCS (estimated from cohorts) |
| **Patient-Reported Outcomes (PROs)** | Hoffmann 2020 (NEI-VFQ25)^21^ | Fibrosis correlated with reduced self-reported visual function and quality of life; CS loss most predictive of PRO decline | Qualitative – worse PRO scores with fibrosis |
| Abbreviations BCVA = Best-corrected visual acuity; ETDRS = Early Treatment Diabetic Retinopathy Study; VA = Visual acuity; MP = Microperimetry; dB = Decibels; LLVA = Low luminance visual acuity; CS = Contrast sensitivity; PROs = Patient-reported outcomes; NEI-VFQ25 = National Eye Institute Visual Function Questionnaire-25 | | | |

## **4. Risk of Bias and Quality Appraisal**

Risk of bias was assessed independently and in duplicate by two reviewers using design-specific, validated instruments. Randomised controlled trials (RCTs) were assessed using the Cochrane Risk of Bias 2.0 (RoB 2) tool, which evaluates five domains: randomisation process, deviations from intended interventions, missing outcome data, measurement of outcomes, and selection of the reported results. Each domain and the overall study were categorised as low risk, some concerns, or high risk, based on the RoB 2 decision algorithm.

Observational studies (cohort, case-control, and interventional case series) were evaluated using the Newcastle–Ottawa Scale (NOS), which assesses the domains of selection, comparability, and outcome/exposure. For comparability, key prespecified confounders included baseline diabetic retinopathy (DR) severity, prior PRP or anti-VEGF status, diabetes duration or HbA1c, and age. Stars were awarded only when these confounders were explicitly adjusted for in design or analysis. Cross-sectional or imaging-only studies were assessed using the adapted NOS for cross-sectional studies, applying analogous criteria for representativeness and reliability of outcome measurement.

To harmonize study-level quality across designs, NOS totals were mapped into three global categories: Low risk: ≥7/9 stars (robust selection, control of major confounders, clear outcome definitions); Moderate risk: 5–6 stars (partial adjustment or minor reporting limitations); High/Serious risk: ≤4 stars (significant confounding, selection, or reporting limitations).

Where both RoB 2 and NOS were applicable (e.g., hybrid or mixed-method studies), the final rating followed the most conservative judgment—that is, the highest risk level across domains or instruments. Disagreements were resolved by consensus, with arbitration by a third senior reviewer when required. Inter-rater agreement for primary domains was high (Cohen’s κ = 0.83).

These risk-of-bias assessments informed sensitivity analyses (restricted to low/moderate-risk studies) and were integrated into GRADE evidence profiles under the risk of bias domain when downgrading overall certainty.

### **Supplemental Table S8.** Risk of bias and quality assessment

Study-level risk of bias is reported by domain and overall rating using RoB 2 for randomized trials (low risk, some concerns, high risk) and the Newcastle–Ottawa Scale for observational studies (stars in selection, comparability, and outcome/exposure; overall category mapped as: ≥7 stars = Low, 5–6 = Moderate, ≤4 = High). Two reviewers independently assessed all studies, with consensus resolution. These ratings were used in prespecified sensitivity analyses and to inform GRADE certainty.

| **Study (Short)** | **Year** | **Participation** | **Attrition** | **Prognostic Factor Measurement** | **Outcome Measurement** | **Confounding** | **Analysis/Reporting** | **Overall Risk** |
| --- | --- | --- | --- | --- | --- | --- | --- | --- |
| **Angermann et al., 2022^36^** | 2022 | Low | Moderate | Moderate | Low | Moderate | Low | Moderate |
| **Barikian et al., 2015^53^** | 2015 | High | Moderate | Moderate | Moderate | High | Moderate | High |
| **Bilgic et al., 2021^54^** | 2021 | Moderate | Moderate | Moderate | Low | Moderate | Moderate | Moderate |
| **Cao et al., 2024^37^** | 2024 | Moderate | Low | Moderate | Low | High | Moderate | Moderate–High |
| **Casalino et al., 2018^35^** | 2018 | Moderate | Moderate | Moderate | Low | Moderate | Low | Moderate |
| **Chakravarthy et al., 2015 (IVAN trial)^19^** | 2015 | Low | Low | Low | Low | Moderate | Low | Low |
| **Chandak et al., 2025 (PRECISE 7)^38^** | 2025 | Low | Low | Low | Low | Moderate | Low | Low |
| **Chandra et al., 2024 (PRECISE Report 2)^68^** | 2024 | Low | Low | Low | Low | Moderate | Low | Low–Moderate |
| **Channa et al., 2015^55^** | 2015 | High | Moderate | Moderate | Moderate | High | Moderate | High |
| **Charles et al., 2023^40^** | 2023 | Moderate | Moderate | Moderate | Low | Moderate | Moderate | Moderate |
| **Cheung et al., 2019^41^** | 2019 | Moderate | Moderate | Moderate | Low | Moderate | Moderate | Moderate |
| **Daniel et al., 2018 (CATT 5-year)^72^** | 2019 | Low | Low | Low | Low | Moderate | Low | Low |
| **Daniel et al., 2019^13^** | 2018 | Low | Low | Low | Low | Moderate | Low | Low |
| **de la Fuente et al., 2024^42^** | 2020 | Low | Low | Low | Low | Low | Low | Low |
| **Dolz-Marco et al., 2017^22^** | 2024 | Moderate | Moderate | Moderate | Moderate | High | Moderate | High |
| **Evans et al., 2020^34^** | 2020 | Moderate | Moderate | Moderate | Moderate | Moderate | Moderate | Moderate |
| **Finn et al., 2021 (CATT)^56^** | 2017 | High | Moderate | Moderate | Moderate | High | Moderate | High |
| **Gianniou et al., 2015^57^** | 2021 | Moderate | Moderate | Moderate | Low | Moderate | Low | Moderate |
| **Gillies et al., 2020^12^** | 2015 | Moderate | Moderate | Moderate | Low | Moderate | Moderate | Moderate |
| **Gonzalez-Buendia et al., 2017^43^** | 2020 | Low | Moderate | Moderate | Low | Moderate | Low | Moderate |
| **Gräfe et al., 2020^52^** | 2017 | Moderate | Moderate | Moderate | Low | Moderate | Moderate | Moderate |
| **Hoffmann et al., 2020^17^** | 2020 | Moderate | Moderate | Low | Low | High | Moderate | Moderate |
| **Ito et al., 2017^70^** | 2020 | Moderate | Low | Moderate | Low | Moderate | Low | Moderate |
| **Jackson et al., 2015 (INTREPID)^58^** | 2017 | Moderate | Moderate | Moderate | Low | Moderate | Moderate | Moderate |
| **Jaffe et al., 2019 (CATT 5-year)^30^** | 2015 | Low | Low | Low | Low | Moderate | Low | Low |
| **Janse van Rensburg et al., 2021^69^** | 2019 | Low | Low | Low | Low | Moderate | Low | Low |
| **Kim et al., 2018^75^** | 2021 | Moderate | Moderate | Moderate | Low | Moderate | Moderate | Moderate |
| **Kim et al., 2020^45^** | 2022 | Moderate | Moderate | Moderate | Low | Moderate | Moderate | Moderate |
| **Kim et al., 2022^44^** | 2018 | Moderate | Moderate | Moderate | Low | Moderate | Moderate | Moderate |
| **Le et al., 2021^71^** | 2020 | Moderate | Moderate | Moderate | Low | Moderate | Moderate | Moderate |
| **Lenhof et al., 2025^25^** | 2021 | High | Moderate | Moderate | Moderate | High | Moderate | High |
| **Lindenberg et al., 2025^46^** | 2025 | Low | Moderate | Moderate | Low | Moderate | Low | Moderate |
| **Liu et al., 2024^59^** | 2025 | Moderate | Moderate | Moderate | Low | Moderate | Low | Moderate |
| **Llorente-González et al., 2022^60^** | 2024 | Moderate | Moderate | Moderate | Low | Moderate | Moderate | Moderate |
| **Maruyama-Inoue et al., 2020^47^** | 2022 | Low | Moderate | Moderate | Low | Moderate | Low | Moderate |
| **Miere et al., 2015^23^** | 2018 | High | Moderate | Moderate | Moderate | High | Moderate | High |
| **Motschi et al., 2021^61^** | 2015 | High | Moderate | Moderate | Low | High | Moderate | High |
| **Ohayon et al., 2020^62^** | 2021 | Moderate | Moderate | Low | Low | High | Moderate | Moderate |
| **Okeagu et al., 2021 (AREDS2)^48^** | 2020 | High | Moderate | Moderate | Moderate | High | Moderate | High |
| **Papavasileiou et al., 2015^63^** | 2021 | Low | Low | Low | Low | Moderate | Low | Low |
| **Querques et al., 2020^18^** | 2015 | High | Moderate | Moderate | Low | High | Moderate | High |
| **Ramtohul et al., 2022^67^** | 2020 | Moderate | Moderate | Moderate | Low | Moderate | Low | Moderate |
| **Roberts et al., 2016^15^** | 2022 | Low | Moderate | Low | Low | Moderate | Low | Moderate |
| **Roberts et al., 2021^24^** | 2016 | Moderate | Moderate | Low | Low | High | Moderate | Moderate–High |
| **Roberts et al., 2022^31^** | 2019 | Moderate | Moderate | Low | Low | High | Low | Moderate |
| **Roberts et al., 2019^32^** | 2021 | Moderate | Moderate | Low | Low | High | Low | Moderate |
| **Romano et al., 2023^8^** | 2022 | Moderate | Moderate | Low | Low | Moderate | Low | Moderate |
| **Romano et al., 2022^73^** | 2022 | Moderate | Moderate | Moderate | Low | Moderate | Moderate | Moderate |
| **Schranz et al., 2024^21^** | 2023 | Low | Moderate | Moderate | Low | Moderate | Low | Moderate |
| **Souied et al., 2020^9^** | 2024 | Moderate | Moderate | Low | Low | High | Low | Moderate |
| **Tan et al., 2024^64^** | 2020 | Low | Moderate | Moderate | Low | Moderate | Low | Moderate |
| **Teo et al., 2020 (FRB!)^49^** | 2024 | Low | Moderate | Moderate | Low | Moderate | Low | Moderate |
| **Teo et al., 2024^50^** | 2020 | Low | Moderate | Moderate | Low | Moderate | Low | Moderate |
| **Toth et al., 2019^33^** | 2019 | Low | Moderate | Low | Low | Moderate | Low | Moderate |
| **Willoughby et al., 2015^16^** | 2015 | Low | Low | Low | Low | Moderate | Low | Low |
| **Wu et al., 2022^65^** | 2022 | Moderate | Moderate | Moderate | Low | High | Moderate | Moderate |
| **Yu et al., 2023 (AVENUE post-hoc)^51^** | 2023 | Low | Low | Low | Low | Moderate | Low | Low |
| **Zhao et al., 2021^66^** | 2021 | Moderate | Moderate | Moderate | Low | Moderate | Moderate | Moderate |

## **5. Certainty of Evidence (GRADE)**

###

### **Supplemental Table S9.** GRADE Evidence Profile

| **Outcome / Question** | **Representative Studies (Designs)** | **Consistency of Results** | **Risk of Bias** | **Indirectness** | **Imprecision** | **Publication Bias** | **Overall Certainty (GRADE)** | **Notes** |
| --- | --- | --- | --- | --- | --- | --- | --- | --- |
| **Impact of fibrosis on vision (BCVA, function, PROs)** | **CATT**: Daniel 2018,^13^ Daniel 2019,^72^ Jaffe 2019^30^ (RCT cohort analyses); **IVAN**: Chakravarthy 2015 (RCT);^19^ **Registry**: Gillies 2020 (FRB!),^12^ Angermann 2022;^36^ **Observational**: Romano 2023,^8^ Querques 2020,^18^ Schranz 2024,^21^ Hoffmann 2020,^17^ Tan 2024,^64^ Okeagu 2021^48^ | High – fibrosis consistently worsens BCVA (−10 to −15 letters) and retinal function (−8–15 dB MP, lower LLVA/CS, worse PROs) | Moderate (fibrosis definitions vary by study) | Direct (all nAMD cohorts) | Low (large N, consistent effects) | Low | Moderate | Most robust evidence; fibrosis strongly associated with worse vision and function |
| **Predictors: SHRM, Type 2 MNV, IRF, SRF** | **SHRM**: Willoughby 2015,^16^ Casalino 2018,^35^ Kim 2018,^75^ Ramtohul 2022,^67^ Liu 2024;^59^  **Type 2 MNV**: Romano 2023,^8^ Souied 2020,^9^ Lenhof 2025,^25^ Liu 2024;^24^  **IRF**: Chandra 2024 (PRECISE),^68^ Roberts 2022,^45^ Lenhof 2025,^25^ Llorente-González 2022;^60^  **SRF**: Gianniou 2015,^57^ Llorente-González 2022,^60^ Chandra 2024^68^ | High – predictors consistent across designs; SRF protective | Moderate (imaging heterogeneity; some retrospective) | Direct | Moderate (some smaller subgroups) | Possible selective reporting | Low | SHRM and Type 2 most robust; SRF consistently protective |
| **Treatment regimen impact (fixed, PRN, T&E)** | **RCTs**: CATT (Daniel 2018,^13^ Jaffe 2019^30^), IVAN (Chakravarthy 2015^19^);  **Registries**: Gillies 2020^12^ (FRB!), Angermann 2022,^36^ Charles 2023^40^ | Moderate – fixed vs PRN little difference; T&E protective if persistent | Low–Moderate (strong RCTs, but registry data retrospective) | Direct | Moderate (adherence variable) | Low | **Moderate** | Undertreatment and non-persistence ↑ fibrosis risk; regimen intensity less critical |
| **Haemorrhage ≥4 DD / BALAD** | Daniel 2018 (CATT 5y),^13^ Ramtohul 2022^67^ | Consistent – large haemorrhage ↑ fibrosis | Low (prospective RCT + longitudinal cohort) | Direct | Moderate (few studies) | Unclear | **Moderate** | Strong predictor but limited evidence base |
| **Fibrosis prevention strategies (early/persistent therapy, anti-fibrotic biomarkers)** | Barikian 2015 (regimen pilot);^53^ de la Fuente 2024 (ML);^42^ Dolz-Marco 2017 (phenotypic regression Type 2→1);^22^ Romano 2023 (10y follow-up);^8^ Souied 2020 (pathways);^9^ Roberts 2019/2022 (PS-OCT biomarkers)^31,32^ | Low – exploratory, inconsistent | Moderate–High (small N, retrospective, modelling bias) | Indirect (not tested in RCTs) | High (wide CIs, exploratory only) | Likely | **Low** | No proven preventive therapy; only signal is early/persistent treatment protective |
| **^Abbreviations^** ^BCVA = Best-corrected visual acuity; PROs = Patient-reported outcomes; MP = Microperimetry; LLVA = Low luminance visual acuity; CS = Contrast sensitivity; SHRM = Subretinal hyperreflective material; MNV = Macular neovascularization; IRF = Intraretinal fluid; SRF = Subretinal fluid; DD = Disc diameter; BALAD = Bacillary layer detachment; RCT = Randomized controlled trial; FRB! = Fight Retinal Blindness! registry; ML = Machine learning; CATT = Comparison of AMD Treatments Trials; IVAN = Inhibition of VEGF in Age-related choroidal Neovascularisation trial.^ | | | | | | | | |

### **Supplementary Table S10– Summary of Findings (SoF) – Subretinal Fibrosis in nAMD**

| **Outcome** | **No. of Studies (Designs)** | **Findings** | **Absolute Effect (Fibrosis vs No Fibrosis)** | **Certainty of Evidence (GRADE)** |
| --- | --- | --- | --- | --- |
| **Impact on vision (BCVA)** | >15 (RCTs: CATT,^13^ IVAN;^19^ Registries: FRB!,^12^ Angermann;^36^ Cohorts: Romano,^8^ Okeagu^48^) | Fibrosis consistently associated with worse VA | ~−10 to −15 ETDRS letters over 2–5 years; up to −16.4 letters at 10 years | **M**oderate |
| **Impact on retinal function (Microperimetry, LLVA, CS, PROs)** | 6 (Querques,^18^ Romano 2022,^73^ Schranz,^21^ Tan,^64^ Hoffmann,^17^ Papavasileiou^63^) | Fibrosis associated with worse retinal sensitivity and functional measures | MP: −8 to −15 dB; CS: −0.3 to −0.5 logCS; PROs worse with fibrosis | **L**ow |
| **Predictors (SHRM, Type 2 MNV, IRF, SRF)** | ~20 (RCT analyses, registries, cohorts) | SHRM and Type 2 MNV ↑ risk; IRF ↑ risk; SRF protective | ORs 2–5 for SHRM/Type 2; OR <1 for SRF protective | **Moderate** |
| **Treatment regimen (Fixed vs PRN vs T&E)** | 10 (CATT,^13,72^ IVAN,^19^ FRB!,^12^ Angermann,^36^ Charles^40^) | Regimen intensity less important than persistence; undertreatment ↑ fibrosis | Non-persistence → 4× fibrosis risk (Angermann); PRN variable outcomes | **Low-Moderate** |
| **Haemorrhage (≥4 DD / BALAD)** | 2–3 (CATT,^13,72^ Ramtohul^67^) | Large haemorrhage consistently ↑ fibrosis risk | HR 2.28 (CATT ≥4 DD); aHR 2.02 (Ramtohul BALAD) | **Low-Moderate** |
| **Prevention strategies (early/persistent therapy, anti-fibrotic biomarkers)** | 5–6 (Barikian,^53^ de la Fuente,^42^ Dolz-Marco,^22^ Romano^8^, Souied,^9^ Roberts^31^) | No proven anti-fibrotic therapy; early/persistent treatment may reduce risk | Exploratory only; ML predictive AUC 0.72 | **Low** |
| **Abbreviations** BCVA = Best-corrected visual acuity; ETDRS = Early Treatment Diabetic Retinopathy Study; MP = Microperimetry; LLVA = Low luminance visual acuity; CS = Contrast sensitivity; PROs = Patient-reported outcomes; SHRM = Subretinal hyperreflective material; MNV = Macular neovascularization; IRF = Intraretinal fluid; SRF = Subretinal fluid; OR = Odds ratio; HR = Hazard ratio; aHR = Adjusted hazard ratio; DD = Disc diameter; BALAD = Bacillary layer detachment; PRN = Pro re nata (as needed); T&E = Treat-and-extend; ML = Machine learning; CATT = Comparison of AMD Treatments Trials; IVAN = Inhibition of VEGF in Age-related choroidal Neovascularisation; FRB! = Fight Retinal Blindness! registry. | | | | |
